# Novel mycoplasma nucleomodulin MbovP475 decreased cell viability by regulating expression of CRYAB and MCF2L2

**DOI:** 10.1080/21505594.2022.2117762

**Published:** 2022-09-19

**Authors:** Gang Zhao, Doukun Lu, Shujuan Wang, Hui Zhang, Xifang Zhu, Zhiyu Hao, Ali Dawood, Yingyu Chen, Elise Schieck, Changmin Hu, Xi Chen, Liguo Yang, Aizhen Guo

**Affiliations:** a National Key Laboratory of Agricultural Microbiology, Huazhong Agricultural University, Wuhan, China; b College of Veterinary Medicine, Huazhong Agricultural University, Wuhan, China; c College of Animal Science and Technology, Hubei Hongshan Laboratory, Huazhong Agricultural University, Wuhan, China; d Key Laboratory of Ruminant Bio-products of Ministry of Agriculture and Rural Affairs, Huazhong Agriculture University, Wuhan, China; e International Research Center for Animal Disease, Ministry of Science and Technology, Huazhong Agricultural University, Wuhan, China; f International Livestock Research Institute, Nairobi, Kenya; g Hubei International Scientific and Technological Cooperation Base of Veterinary Epidemiology, Huazhong Agricultural University, Wuhan, China

**Keywords:** Mycoplasmas, nucleomodulin, *mycoplasma bovis* MbovP475, pathogenesis, CRYAB, MCF2L2, cell viability

## Abstract

Nucleomodulins are secreted bacterial proteins whose molecular targets are located in host cell nuclei. They are gaining attention as critical virulence factors that either modify the epigenetics of host cells or directly regulate host gene expression. *Mycoplasma bovis* is a major veterinary pathogen that secretes several potential virulence factors. The aim of this study was to determine whether any of their secreted proteins might function as nucleomodulins. After an initial *in silico* screening, the nuclear localization of the secreted putative lipoprotein MbovP475 of *M. bovis* was demonstrated in bovine macrophage cell line (BoMac) experimentally infected with *M. bovis*. Through combined application of ChIP-seq, Electrophoretic mobility shift assay (EMSA) and surface plasmon resonance (SPR) analysis, MbovP475 was determined to bind the promoter regions of the cell cycle central regulatory genes *CRYAB* and *MCF2L2*. MbovP475 has similar secondary structures with the transcription activator-like effectors (TALEs). Screening of various mutants affecting the potential DNA binding sites indicated that the residues ^242^NI^243^ within MbovP475 loop region of the helix-loop-helix domain were essential to its DNA binding activity, thereby contributing to decrease in BoMac cell viability. In conclusion, this is the first report to confirm *M. bovis* secretes a conserved TALE-like nucleomodulin that binds the promoters of *CRYAB* and *MCF2L2* genes, and subsequently down-regulates their expression and decreases BoMac cell viability. Therefore, this study offers a new understanding of mycoplasma pathogenesis.

## Introduction

Mycoplasmas are the smallest free-living wall-less prokaryotic pathogens with minimal genomes. They belong to the class *Mollicutes*, which contains many pathogenic species infecting animals and humans [1], and might have evolved from gram-positive ancestors by severe genome reduction, leading to the exclusion of the classical repertoire of virulence genes in pathogenic mycoplasmas. The ability to cause both persistent infection and severe debilitating disease in humans and many species of animals has been retained. The recent increase in antibiotic resistance and decrease in response to chemotherapy has caused growing concern about pathogenic mycoplasmas in both medical and veterinary fields [2]. However, the lack of understanding of their pathogenesis has impeded the development of highly effective measures against mycoplasmosis.

Previous studies have identified some of the genes involved in optimal adhesion, immune evasion, or immunomodulation, and suggested that efficient nutrient scavenging might be involved in virulence. In addition, exacerbated inflammation might cause tissue damage [3]. The cytotoxic products, such as hydrogen peroxide, and polysaccharides may be important for the virulence of some mycoplasma species such as *Mycoplasma mycoides subsp. mycoides* SC (MmmSC) [4–6]. Until now, most mycoplasma virulence studies have focused on surface proteins, such as the wide array of variable surface lipoproteins (Vsps) [7], membrane proteins, which may function as adhesins [8–12], or stimulators of the host immune system [13–15], and membrane-associated enzymes, such as nucleases that regulate pathogenicity and cytotoxicity [16–19]. Secretome studies of some mycoplasma species such as *M. pneumoniae*, *M. synoviae*, *Acholeplasma laidlawii*, and *M. bovis* have predicted many and confirmed some secreted proteins in mycoplasmas through a LC-MS/MS proteomic approach [20–23]. However, to determine the functions of these proteins is difficult because most of them are putative proteins, and efficient genetic tools and animal infection models for confirmation are lacking. To date, the functions of only a small portion of mycoplasma secreted proteins have been demonstrated, such as P80 of *M. hominis* [24], the Community Acquired Respiratory Distress Syndrome (CARDS) toxin [25], and Mpn491 nuclease [26] of *M. pneumoniae*, the apoptosis inducer MbovP280 of *M. bovis* [27], and secreted serine protease S41 of *M. capricolum* [28].

One of the critical strategies among pathogenic bacteria involves translocation of their secreted effector proteins into host cells for the modulation of host biological processes. Some proteins are translocated into nuclei of host cells. These nuclear-targeting proteins can be categorized as either nucleomodulins, which have epigenetic-modulating activities, or cyclomodulins, which specifically interfere with the host cell cycle. However, both are generally considered as nucleomodulins because they usually have multiple functions thereby functioning as important virulence factors [29]. For example, they may regulate the expression of host genes associated with immune response, proliferation, and apoptosis [30], by binding to the promoter of host genes; and the nuclear histones to modify epigenetics probably via methylation, acetylation, and ubiquitination; or binding to the epigenetic regulators, transcription or splicing factors, and signalling proteins. To date, at least 70 nucleomodulins have been reported in bacterial pathogens infecting humans, animals, and plants [29]. The transcription activator-like effectors (TALEs) are a type of nucleomodulins first found in the *Xanthomonas* genus of plant pathogenic bacteria; these nucleomodulins regulate target genes in hosts through the interaction between their DNA-binding modules and their host target DNA sequences [31]. In mycoplasmas, the only identified nucleomodulins to date are three secreted DNA methyltransferases of *M. hyorhinis*, which have been found to promote tumorigenesis of cultured human cells by binding GC and GATC specific motif of DNA in the cellular target genes, and catalysing DNA methylation [32]. Other Mollicutes species, such as Aster Yellows phytoplasma, have been shown to secrete nucleomodulin SAP11, which binds and destabilizes transcription factors that control plant development and promote the expression of lipoxygenase genes [33].

*M.bovis* causes pneumonia, as well as other serial diseases such as mastitis and arthritis, thus resulting in large economic losses in the cattle industry worldwide [34]. Our previous investigation has revealed the secretome of *M. bovis* through an LC-MS/MS proteomic approach [22,35]. Several secreted proteins have been confirmed to have virulence related activities, such as stimulating apoptosis [27], inflammation, and cytotoxicity as a secreted nuclease [16]. However, whether *M. bovis* secretes any nucleomodulins is unknown yet. Therefore, in this study, we aimed to use *M. bovis* as a model to identify novel nucleomodulins secreted by mycoplasmas, and assess their functions in regulating gene expression of host cells. Among the secretome of *M. bovis*, MbovP475 was confirmed to be a secreted protein with the high abundance [27]. In this study, a series of experiments at the molecule and whole cell levels demonstrated MbovP475 is a TALEs-like nucleomodulin that binds DNA sequences of its nuclear targets. These findings represent a breakthrough in the understanding of mycoplasma pathogenesis.

## Results

### Confirmation of M. bovis nucleomodulins with TALEs-like domain

**Figure 1.**
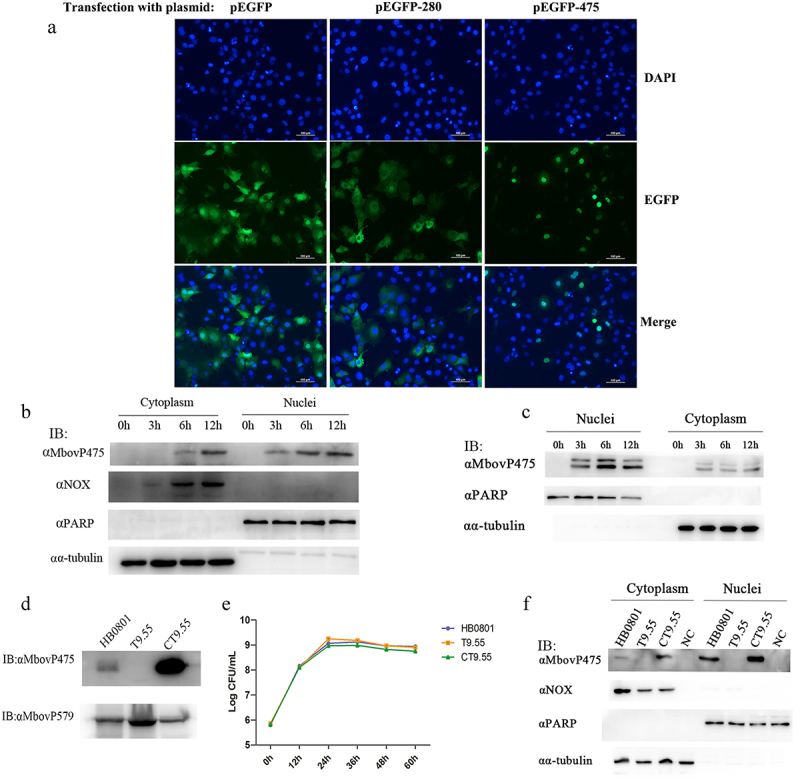
The nuclear localization of MbovP475. (a) the localization of MbovP475 encoded by pEGFP-475 plasmid in BoMac was detected by fluorescence microscopy at 24 h post-transfection. The cells transfected with pEGFP-280 served as negative control. (b, c) Nuclear location of MbovP475 was verified by western blotting assay. The cell fractions of BoMac were extracted and resolved with SDS-PAGE at 3, 6 and 12 h post-infection with *M. bovi*s or treatment with rMbovp475. Then, the proteins were transferred onto PVDF membrane and immunodetected with the antibody directed against MbovP475. NOX served as marker for *M. bovis*. α-tubulin and PARP served as markers for the cytosolic and nuclear fractions, respectively. (d) Visualization of MbovP475 expression in T9.55 (MbovP475 knock-out mutant) and CT9.55 (MbovP475 complemented strains) with western blotting assay. Wild-type strain HB0801 was used as the positive control. (e) Growth curves of HB0801, T9.55, and CT9.55 strain. Growth of *M. bovis* at each time point was determined with a plating assay. (f) the nuclear localization of MbovP475 in BoMac at 12 h post-infection with *M. bovis* strains was verified by western blotting assay, as described above.

On the basis of the nuclear localization signals (NLS) and tandem repeats characterized by the TALEs-domain, SeqNLS and BioAider V1.334 predicted 11 TALEs-like nucleomodulins of *M. bovis*: MbovP145, MbovP290, MbovP339, MbovP467, MbovP475, and six variable surface proteins (Vsps) MbovP793, MbovP794, MbovP795, MbovP796, MbovP797, and MbovP798 (Table 1). Six proteins including five non-vsp proteins MbovP145, MbovP290, MbovP339, MbovP467, and MbovP475, and one representative Vsp MbovP796 were selected to confirm their subcellular location in the host cells, while the known secreted but cytoplasmic MbovP280 [27] was used as the negative control. We labelled the proteins with enhanced green fluorescent protein, and constructed corresponding recombinant plasmids pEGFP-145, pEGFP-290, pEGFP-339, pEGFP-467, pEGFP-475, pEGFP-796, and pEGFP-280, which were transfected into bovine macrophage cell line (BoMac) cells together with the pEGFP vector and observed under a fluorescence microscope. Punctuated green fluorescence only presented in the nuclei of cells transfected with pEGFP-475, whereas in cells transfected with pEGFP-280, diffuse green fluorescence presented in the cytoplasm as expected (Figure 1(a)). The BoMac cells transfected with the recombinant plasmids encoding other five predicted TALEs-like nucleomodulins showed diffuse green fluorescence in the cellular cytoplasm (Fig. S1).

**Table 1.** The prediction of TALE-like nucleomodulins in *M. bovis.*

Proteins	Predicted NLS	Start	Stop	Score	Tandem repeats
MbovP145	REHKYNNDKTK	141	151	0.536	contain
MbovP290	KKLFKDAKANKNKKYKE	236	252	0.884	contain
MbovP339	KKSK	2	5	0.874	contain
MbovP467	KKQNVTELEKEIKRLRN	112	128	0.884	contain
MbovP475	KRKFSLL	2	8	0.644	contain
MbovP793	KKSK	2	5	0.879	contain
MbovP794	KKSK	2	5	0.878	contain
MbovP795	KKSK	2	5	0.879	contain
MbovP796	QNTPIKK	185	191	0.755	contain
MbovP797	SSKKDKDPKTESS	244	256	0.887	contain
MbovP798	KKSK	2	5	0.879	contain

The MolliGen 3.0 web server was used to identify homologous proteins of MbovP475. MbovP475 has a homolog in *Mesoplasma florum* and eight ruminant infecting mycoplasma species: *M. bovis, M. feriruminatoris*, *M. agalactiae*, *M. capricolum subsp. capricolum*, *M. mycoides subsp. capri*, *M. putrefaciens*, *M. leachii*, and *M. mycoides subsp. mycoides* (with an E value less than e^−8^ indicating it is conservative in ruminant mycoplasmas (Fig. S2).

### Confirmation of nuclear location of MbovP475

To further verify the nuclear location of MbovP475 in *M. bovis* infected cells, BoMac cells were infected with *M. bovis* HB0801 at a multiplicity of infection (MOI) of 500. The cytoplasmic and nuclear proteins were extracted at 0, 3, 6, and 12 h post-infection and subjected to western blotting assay by using cellular nucleic protein PARP and cytoplasmic protein α-tubulin and *M. bovis* protein NADH oxidase (NOX) as the controls. As expected, the PARP was located in only the nuclei, whereas α-tubulin presented in only the cytoplasm from 0 to 12 h post-infection. Meanwhile, *M. bovis* NOX presented in the cytoplasm of the BoMac cells at 6 and 12 h post-infection, and its presence increased with time. However, although MbovP475 presented in both nuclei and cytoplasm, it was preferentially located in the nuclei (at 3, 6, and 12 h post-infection) and was observed at one timepoint earlier in the nuclei than the cytoplasm (at 6 and 12 h post-infection; Figure 1B). To detect the internalization of extracellular MbovP475, we used the recombinant protein rMbovP475 previously prepared by our laboratory [27] to treat BoMac cells: 10 μg rMbovP475 was incubated with BoMac cells in one well containing 1 mL medium for 12 h. Similar to *M. bovis* infection, western blotting assay showed that rMbovP475 presented in both nuclei and cytoplasm between 3 and 12 h after treatment, and more rMbovP475 was observed in the nuclei. The extra band of the rMbovP475 might be caused by the tag cleavage (Figure 1(c) and Fig. S3).

The Mbov_0475 gene knock-out mutant T9.55 was identified from the transposon-mediated *M. bovis* mutant library previously prepared in our laboratory [11], and its complement strain CT9.55 was constructed in this study. Western blotting assays with the wild-type HB0801, T9.55, and CT9.55 strains and the recombinant membrane protein rMbovP579 used as the control confirmed that MbovP475 expression was deficient in T9.55 but presented in both HB0801 and CT9.55 (Figure 1(d)). However, this differential expression of MbovP475 did not affect the growth phenotype of three *M. bovis* strains in cell free PPLO-medium (Figure 1(e)). Furthermore, BoMac cells were infected with these three strains HB0801, T9.55, and CT9.55 for 12 h, and the subcellular localization of MbovP475 was assessed with western blotting assays. The findings further verified that MbovP475 presented in both the nuclei and cytoplasm of HB0801 and CT9.55 infected BoMac cells, but not T9.55 infected cells (Figure 1(f)).

Together, our findings demonstrated that MbovP475, either secreted naturally by *M. bovis* or in the form of recombinant protein, entered the nuclei of *M. bovis* infected or rMbovP475 treated BoMac cells.

### Determination of the NLS in MbovP475

**Figure 2.**
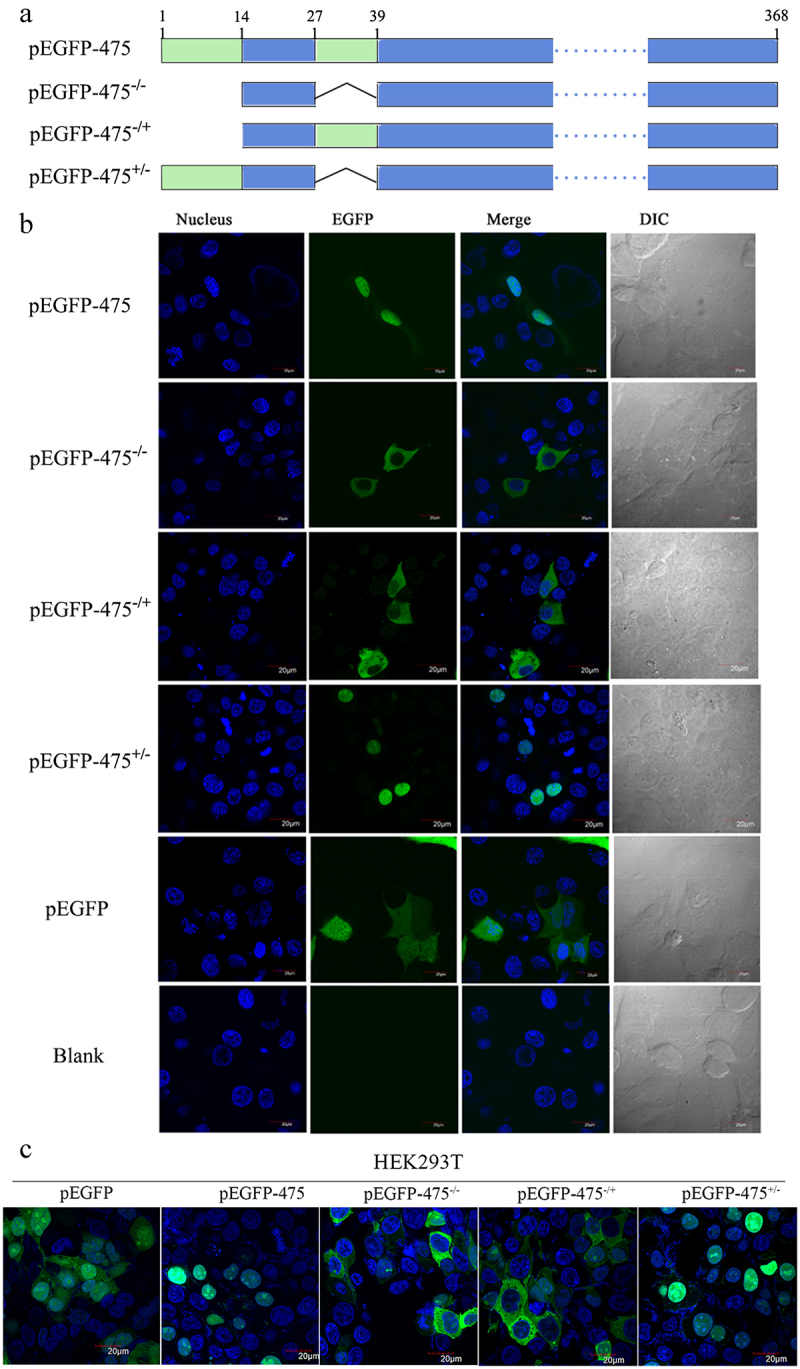
The aa 1–13 fragment of MbovP475 functions as a nuclear localization signal. (a) the schematic diagram shows the MbovP475 deletion in various constructs used for transfection. The aa 1–13 (green box) and aa 28–38 (green box) fragments were predicted as NLS by SeqNLS. plasmids pEGFP-475, pEGFP-475^−/−^, pEGFP-475^−/+^, and pEGFP-475± express full length MbovP475, truncated MbovP475 missing aa 1–13 and aa 28–38, truncated MbovP475 missing aa 1–13, and truncated MbovP475 missing aa 28–38 respectively. (b) the localization of MbovP475 in Bomac cells was detected by confocal microscopy at 24 h post-transfection with the above plasmids. (c) the localization of MbovP475 in HEK293T was detected by confocal microscopy at 24 h post-transfection with the above plasmids. The cells transfected with pEGFP-280 served as negative control. The aa 1–13 fragment is essential for MbovP475 to enter the nucleus of BoMac. pEGFP and blank mean cells transfected with empty vector and PBS respectively.

Because the SeqNLS webserver predicted that MbovP475 had two NLS corresponding to the fragments of aa 1–13 and aa 28–38 at the *N*-terminal region, we had to determine the functional NLS. The plasmids encoding intact MbovP475 gene or truncated MbovP475 genes lacking one or both NLS which were fused with EGFP gene were constructed and designated as the pEGFP-475^−/+^ with deletion of aa 1–13, pEGFP-475± with deletion of aa 28–38, and pEGFP-475^−/−^ with deletion of both NLS fragments; the pEGFP blank vector was used as the negative control (Figure 2(a)). These plasmids were then transfected into BoMac and HEK293T cells. Only the pEGFP-475 and pEGFP-475^+/-^ transfected cells showed nuclear green fluorescence, whereas the others showed cytoplasmic green fluorescence, thus indicating that the functional NLS of MbovP475 was aa 1–13 (Figure 2(b) and 2C and Fig. S4).

### Inhibitory effect of MbovP475 on BoMac cell viability

**Figure 3.**
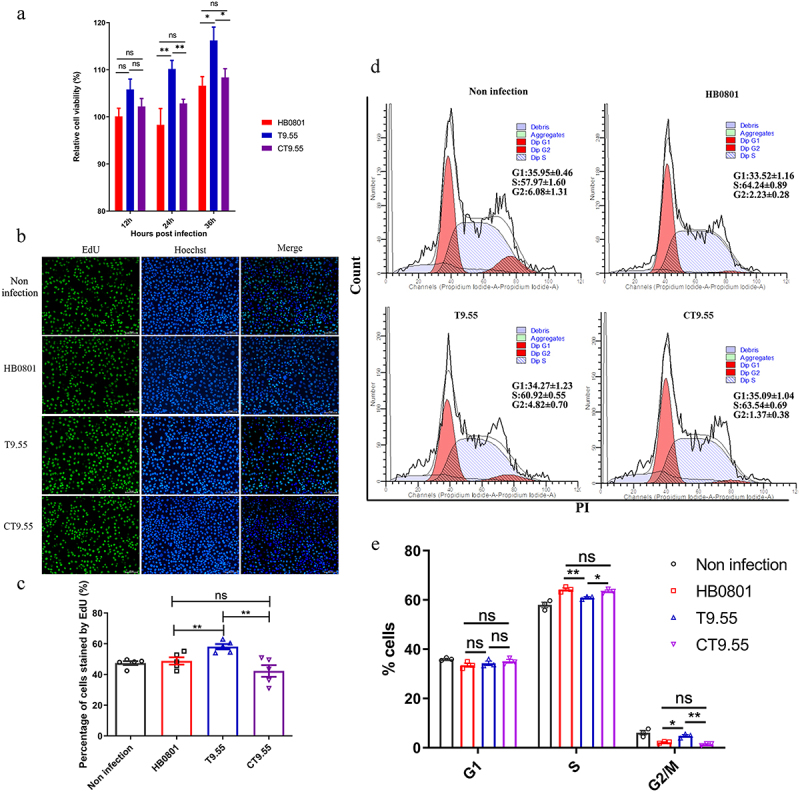
MbovP475 decreased BoMac cell viability. (a) the relative cell viability of BoMac infected with *M. bovis* was detected by CCK-8. BoMac cells infected with *M. bovis* HB0801, T9.55, or CT9.55 strains at an MOI of 5 for 12 h, 24 h, and 36 h. (b, c) the cell proliferation of BoMac infected with *M. bovis* at an MOI of 5 for 24 h was detected by EdU assay. The nuclei of proliferative cells were stained with EdU (green), and the nuclei of all cells stained with Hoechst (blue). The percentage of cells stained by EdU of each group (mean and SEM, n = 5) was calculated by Image-Pro plus 6.0 software. (d, e) the cell cycle of BoMac infected with *M. bovis* at an MOI of 5 for 24 h was detected by flow cytometry. The DNA content of cells was stained with PI and detected. The percentage of cells in G1 phase, S phase, and G2/M phase of each group was calculated and compared (mean and SEM, n = 3). The data of a and E were analysed with one way ANOVA and Tukey’s test while data of c with Two-tailed Student’s *t*-test. *p <0.05 and **p <0.01 indicate statistically significant differences; ns indicates no difference.

To explore the effect of MbovP475 on cell viability during *M. bovis* infection, we analysed the cell viability of BoMac cells infected with the strains HB0801, T9.55 (MbovP475 knock-out mutant) and CT9.55 (MbovP475 complementary strains). The results indicated that the BoMac cells infected with T9.55 had significantly higher relative cell viability than those infected with either HB0801 or CT9.55 at 24 h (*p* < 0.01) and 36 h (*p* < 0.05) post-infection, whereas no difference in relative cell viability was observed between HB0801 and CT9.55 (*p* > 0.05; Figure 3(a)). Apoptosis detection of BoMac cells treated with rMbovP475 revealed that rMbovP475 did not induce apoptosis (Fig. S5).

To demonstrate the mechanism underlying MbovP475’s inhibitory effect on cellular viability, we evaluated the proliferation of BoMac cells infected with the above three *M. bovis* strains through 5-ethynyl-29-deoxyuridine (EdU) assay. The intensity of Alexa Fluor 488 and Hoechst 33,342 in infected BoMac cells was quantified with Image-Pro plus 6.0 software and the results revealed that BoMac proliferation was inhibited by both HB0801 and CT9.55 infection compared with T9.55 infection (undefinedFigure 3(b,c)) (*p* < 0.01). The cell cycle was detected with flow cytometry after infection with these three strains, and 10,000 cells in each group were counted. The proportions of BoMac cells infected with HB0801 (64.24% ± 0.89%) and CT9.55 (63.54% ± 0.69%) in S phase were significantly higher than those of the cells infected with T9.55 (60.92% ± 0.55%) and PBS (57.97% ± 1.60%; *p* < 0.05), whereas the percentage of the cells infected with HB0801 (2.32% ± 0.28%) and CT9.55 (1.37% ± 0.38%) in G2/M phase were significantly lower than those of the cells infected with T9.55 (4.82% ± 0.70%) and PBS (6.08% ± 1.31%; *p* < 0.05; undefinedFigure 3(d,e)). These results revealed that MbovP475 increased the proportion of cells in S phase and decreased the proportion of cells in G2/M phase, indicating S phase arrest of HB0801 and CT9.55 infected cells occurred. Together, our results demonstrated that *M. bovis* MbovP475 decreased the cell viability of BoMac.

### ChIP-Seq analysis of MbovP475-binding genes

To enrich more MbovP475-binding DNA fragments, we infected BoMac cells with *M. bovis* at MOI of 500. Then ChIP-seq analysis was performed to explore the potential MbovP475-binding sites in BoMac genomic DNA. By comparing the Input-DNA before enrichment with antibody with fold enrichment exceeding 1.5, we identified a total of 2648 potential MbovP475-binding sites (Supplementary Data 1). Of these, 72.06% were localized in intergenic regions, 18.46% were localized in intronic regions, 1.68% were localized in exon regions, and 7.82% were localized in other regions (Fig. S6A). Further, the most of MbovP475-binding peaks were concentrated within 2 kb around the transcription start site (TSS) of the nearest protein-coding genes or ncRNA (Fig. S6B). Therefore, the next analysis focused on the 194 MbovP475-binding peaks within 2 kb from the TSS (Supplementary Data 2). The distribution of those peaks on chromosomes showed high enrichment in chromosome X and 2 (Fig. S6C). On the basis of the peak sequences in the MbovP475-binding regions, MEME motif analysis was performed. The most probable consensus motif is shown in Fig. S6D. The consensus motif was found in the promoter region of CRYAB and MCF2L2 (Fig. S6E). KEGG pathway enrichment analysis demonstrated that top 20 enrichment pathways included Ribosome, Lysine degradation, Long-term depression, etc (Fig. S6F). The cell cycle pathway was confirmed to be presented in the list of top 20 enrichment pathways. These results suggested that MbovP475 would bind the promoter regions of the target genes CRYAB and MCF2L2 in the nuclei of infected BoMac cells.

### Verification of MbovP475-regulated genes

**Figure 4.**
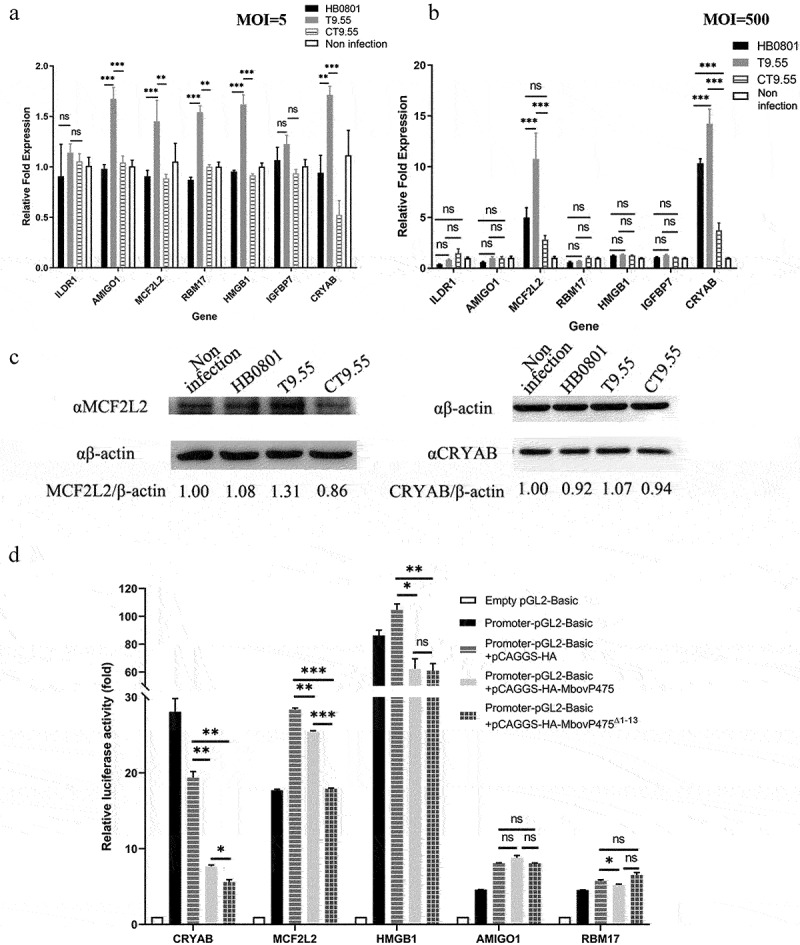
Transcriptional regulation of target genes by MbovP475. (a) the mRNA levels of ILDR1, AMIGO1, MCF2L2, RBM17, HMGB1, IGFBP7, and CRYAB in BoMac at 12 h post-infection with *M. bovis* (MOI = 5) were detected by RT-PCR. (b) the mRNA levels of above target genes in BoMac at 12 h post-infection with *M. bovis* (MOI = 500) were detected by RT-PCR. The data of a and B (mean and SEM; n = 4) were analysed with one way ANOVA and Tukey’s test, *p <0.05, **p <0.01, ***p <0.001 indicate statistically significant differences; ns indicates no difference. (c) the expression of CRYAB and MCF2L2 in BoMac at 24 h post-infection with *M. bovis* (MOI = 5) was detected by western blotting assay. (d) Luciferase reporter assay on transcription-regulating activity of potential target genes in HEK293T cells by MbovP475. The cells co-transfected with 1 μg promoter-pGL3-Basic and 1 μg pCAGGS vector encoding HA, HA-MbovP475, or HA-MbovP475^Δ1-^^13^. the empty pGL2-Basic vector was used as the negative control. Resultant luciferase activities were expressed as relative luciferase activities normalized to the pRL-TK activity and shown as mean ± SEM from three independent experiments. Two-tailed Student’s *t*-test was used, *p <0.05, **p <0.01, ***p <0.001 indicate statistically significant differences; ns indicates no difference.

To further explore the possible regulation of cell viability through the interaction between MbovP475 and cellular DNA, we selected seven host genes from the 194 genes associated with cell proliferation and their MbovP475-binding peaks within 2 kb of the TSS in host genes (Supplementary Data 2) for verification: CRYAB [36], ILDR1 [37], MCF2L2 [38], AMIGO1 [39], HMGB1 [40], IGFBP7 [41], and RBM17 [42] (Table 2). Their gene transcription in BoMac cells infected with *M. bovis* at the MOI of 5 was examined with RT-PCR at 12 h post-infection. The results indicated that AMIGO1, MCF2L2, RBM17, HMGB1, and CRYAB genes were significantly upregulated in T9.55 infected cells compared with HB0801 and CT9.55 infected cells, thus indicating that MbovP475 inhibited their transcription (*p* < 0.01; Figure 4(a)). However, when MOI was increased to 500, only the transcription of MCF2L2 and CRYAB genes in T9.55 infected cells was further increased (*p* < 0.001; Figure 4(b)), thereby demonstrating that MbovP475 primarily correlated with the suppression of MCF2L2 and CRYAB transcription at both high and low levels of MOI. The transcription of MCF2L2 and CRYAB in the cells infected with high MOI was higher than that of MCF2L2 and CRYAB in the cells infected with low MOI of T9.55. This means the expression of selected genes was not only regulated by MbovP475, but also by other components of *M. bovis*. Western blotting assay further verified that deletion of MbovP475 in T9.55 increased the expression of CRYAB and MCF2L2 above that in HB0801 and CT9.55 (Figure 4(c), and Fig. S7).

**Table 2.** The screening of proliferation associated genes potential regulated by MbovP475.

Gene stable ID	Gene name	**Distance** (Summit of peaks from TSS)	Reference
ENSBTAG00000000434	*CRYAB*	+138	[36]
ENSBTAG00000004705	*ILDR1*	−210	[37]
ENSBTAG00000010394	*MCF2L2*	+83	[38]
ENSBTAG00000014083	*AMIGO1*	−80	[39]
ENSBTAG00000014862	*HMGB1*	+178	[40]
ENSBTAG00000019368	*IGFBP7*	+188	[41]
ENSBTAG00000021951	*RBM17*	+144	[42]

To elucidate the regulatory mechanism of MbovP475, the promoter regions of CRYAB, MCF2L2, HMGB1, AMIGO1, and RBM17 genes were inserted into PGL3-basic vector to construct a series of reporter plasmids. HEK293T cells were, respectively, co-transfected with those reporter plasmids and the pCAGGS plasmids (Table S1) encoding HA tag, HA-MbovP475, or HA-MbovP475^Δ1-^^13^. Both MbovP475 and HA-MbovP475^Δ1-^^13^ significantly suppressed the transcription activity, as shown by the relative luciferase activity induced by the promoters of CRYAB, MCF2L2, and HMGB1(Figure 4(d)). Overall, MbovP475 suppressed the expression of CRYAB and MCF2L2 genes by interacting with their promoters.

### Reduction of CRYAB and MCF2 L expression depending on MbovP475 binding to their promoters

**Figure 5.**
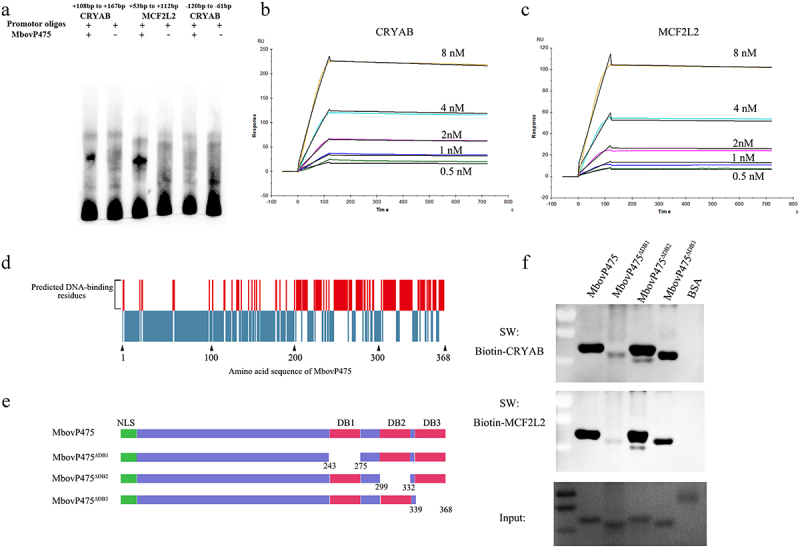
The verification of interaction between MbovP475 and promoter fragments. (a) EMSA analysis of the interaction between MbovP475 and the promoter region of MCF2L2 (+53 bp to +112bp from TSS) or CRYAB (+108 bp to + 167 bp from TSS) using 5 μg MbovP475 incubated with 50 fmol promoter fragment of target genes. The CRYAB promoter region (−120 bp to − 61 bp from TSS) served as negative control to demonstrate specific binding. (b, c) Determination of the affinity and kinetics of molecular interaction by SPR. About 100 RU MbovP475 captured on a charged NTA sensor chip and test promoter fragments at five concentrations between 0.5 and 8 nM. (d) the amino acid residue positions of MbovP475 potentially involved in interaction with the promoter fragment were predicted by DP-bind (red bars). (e) the schematic diagram shows the truncation of MbovP475 used in Southwestern blotting assay. The MbovP475^∆db1^, MbovP475^∆db2^, and MbovP475^∆db3^ represent the truncated MbovP475 missing the predicted DNA-binding amino acid residues. (f) Identification of DNA-binding region of MbovP475 by Southwestern blotting assay. 2 μg of MbovP475, MbovP475^∆db1^, MbovP475^∆db2^, and MbovP475^∆db3^ were resolved with SDS-PAGE and transferred to nitrocellulose membranes. The samples were then incubated with 20 pmol biotin labelled promoter fragments at room temperature for 12 h after renaturation in TNED buffer.

To determine whether MbovP475 directly bound the promoters of its target genes CRYAB and MCF2L2 and regulated their transcription, the interaction between rMbovP475 and the promoter fragments was checked with EMSA. Considering that the summit of MbovP475-binding peaks was located at + 138 bp from TSS of CRYAB and +83 bp from TSS of MCF2L2 (Table 2 and Fig. S6E), we synthesized the DNA fragments of CRYAB (+108 bp to + 167 bp) in Chromosome 15 and MCF2L2 (+53 bp to + 112 bp) in Chromosome 1 for EMSA. Other fragment of CRYAB promoter (−120 bp to −61 bp) served as the negative control. As a result, the MCF2L2 promoter fragment (+53 bp to + 112 bp) and CRYAB promoter (+108 bp to + 167 bp) directly bound to MbovP475, whereas the negative control fragment of CRYAB promoter (−120 bp to −61 bp) did not bind to MbovP475 (Figure 5(a) and Fig. S8). The SPR assay was further applied to determine the affinity and kinetics of molecular interaction. In agreement with the above findings, these two fragments were identified as the potential hits at five concentrations between 0.5 nM and 8 nM. The affinities between MbovP475 and the promoter fragments of CRYAB and MCF2L2 was 1.73 × 10^−10^ M and 1.04 × 10^−10^ M, respectively (Figure 5(b,c)).

**Figure 6.**
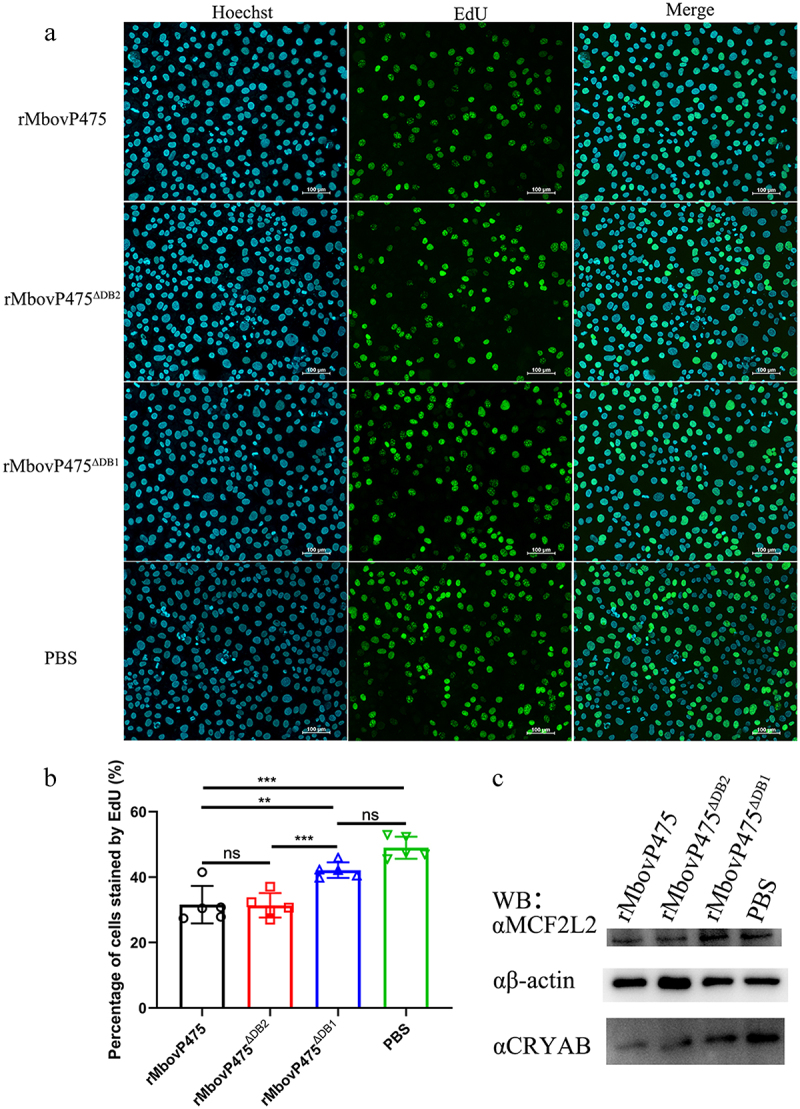
Confirmation of DB1 domain essential for MbovP475’s inhibition of cell proliferation. (a) the cell proliferation of BoMac treated with 1 μM rMbovp475, rMbovp475^ΔDB1^, and rMbovp475^ΔDB2^ for 24 h was detected with EdU assay. Cells treated with PBS served as negative control. The nuclei of proliferative cells stained with EdU (green), and the nuclei of all cells stained with Hoechst (blue). (b) the percentage of cells stained by EdU in each group (mean and SEM, n = 5) was calculated in Image-Pro plus 6.0 software. Two-tailed Student’s *t*-test was used, *p <0.05, **p <0.01, ***p <0.001 indicate statistically significant differences; ns indicates no difference. (c) the expression of MCF2L2 and CRYAB in the cells treated with 1 μM rMbovp475, rMbovp475^ΔDB1^, and rMbovp475^ΔDB2^ for 24 h. The cells treated with PBS served as negative control.

The DNA-binding residues in MbovP475 were predicted by DP-bind software (Figure 5(d)). Then, based on the prediction, three truncated mutants of MbovP475 were successfully constructed as follows and expressed: MbovP475^∆DB1^ with deletion of aa 243–275, MbovP475^∆DB2^ with deletion of aa 299–332 aa, and MbovP475^∆DB3^ with deletion of aa 339–368 (Figure 5(e)). The interaction between the purified truncated rMbovP475 proteins and the wild-type rMbovP475 and either biotin labelled CRYAB promoter fragment (+108 bp to + 167 bp) or biotin labelled MCF2L2 promoter fragment (+53 bp to + 112 bp) was determined by Southwestern blotting assay. The negative control BSA showed no binding to both promoter fragments; MbovP475^∆DB1^ lost most of its binding to CRYAB and MCF2L2 promoters; however, MbovP475^∆DB2^ and MbovP475^∆DB3^ showed no changes in binding ability (Figure 5(f) and Fig. S8). Therefore, these results showed that MbovP475 directly bound to the promoters of CRYAB and MCF2L2 through the DNA binding domain at DB1 between aa 243 and 275 in MbovP475. To reveal the association between downregulation of target gene expression and interaction between target genes and MbovP475, we treated cells with 1 μM rMbovP475, rMbovP475^ΔDB1^, rMbovP475^ΔDB2^, and equal volume of PBS as the blank control respectively. EdU assay revealed that rMbovP475 and rMbovP475^ΔDB2^, as compared with rMbovP475^ΔDB1^ and PBS, significantly inhibited the cell proliferation (*p* < 0.01; undefinedFigure 6(a,b)). The expression of CRYAB and MCF2L2 in cells treated with above proteins was detected by western blotting assay. The results indicated that rMbovP475 and rMbovP475^ΔDB2^, as compared with rMbovP475^ΔDB1^ and PBS, inhibited the expression of CRYAB and MCF2L2 (Figure 6(c) and Fig. S8). These results demonstrated the DB1 domain is essential for MbovP475’s DNA binding and subsequently suppressing the expression of two target genes

**Figure 7.**
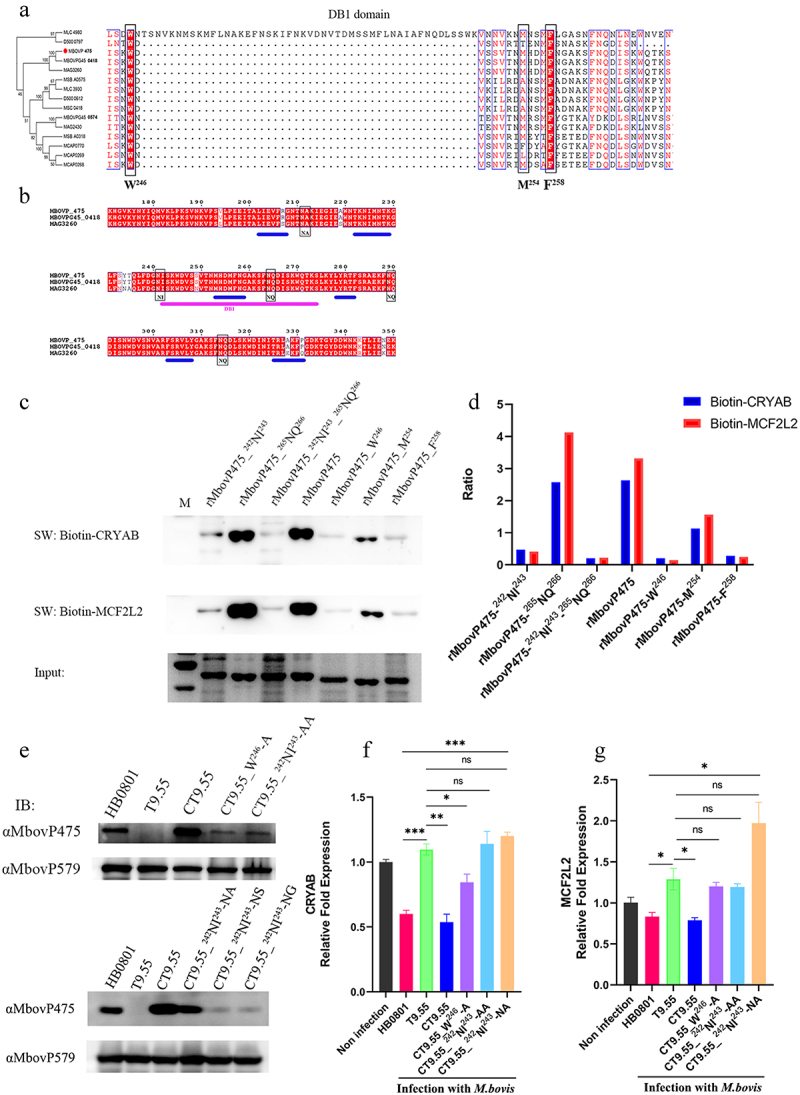
Determination of DNA-binding sites of MbovP475. (a) the analysis on conserved amino acid residues of MbovP475 DB1 domain with MolliGen. The conserved amino acid residues were marked with black boxes. (b) the secondary structure analysis on MbovP475 DB1 domain with Jpred 4. The predicted DNA-binding residues in the loop region between two-helix were marked with black boxes. (c) the interaction between mutated MbovP475 and DNA. 2 μg MbovP475 and its mutants were resolved with SDS-PAGE and transferred to nitrocellulose membranes and incubated with 20 pmol biotin labelled promoter fragments at room temperature for 12 h. Then the bands were detected by Streptavidin-Horseradish Peroxidase Conjugate. (d) the relative ratio of the integrated density value of band on the membrane to the integrated density value of band on the polyacrylamide gel. (e) the expression of mutated MbovP475 in complementary strains was detected by western blotting assay. The MbovP579 served as control. The expression of CRYAB (f) and MCF2L2 (g) in BoMac at 12 h post-infection with *M. bovis* (MOI = 5) was detected by qRT-PCR. Results were normalized to β-actin mRNA and shown as mean ± SEM from three independent experiments. Two-tailed Student’s *t*-test was used, *p <0.05, **p <0.01, ***p <0.001 indicate statistically significant differences; ns indicates no difference. (f, g; mean and SEM; n = 3).

The DNA-binding residues in DB1 region of MbovP475 were further explored by analysis of conserved residues among homologous sequences and secondary structures. Multiple sequence alignments revealed that the most conserved amino acid residues were tyrosine at the aa 246 site and phenylalanine at the aa 258 site (Figure 7(a)). Secondary structure analysis indicated that MbovP475 has five tandem amino acid repeats containing six helixes characterized by TALEs, which uses the loop region between two-helix bundles to bind DNA [43]. The predicted DNA-binding residues are marked in the black box in Figure 7(b). The DNA binding ability of the purified rMbovP475 mutants whose conserved amino acid residues and predicted DNA-binding residues, as mentioned above, were replaced by alanine, was evaluated by using the wt rMbovP475 as the positive control in the Southwestern blotting assay. The combined mutation of N242A and I243A (rMbovP475-^242^NI^243^) and the single mutations (W246A (rMbovP475-W^246^) and F258A (rMbovP475-F^258^), almost completely removed binding of MbovP475 to the promoter fragments of CRYAB and MCF2L2. The mutation M254A (rMbovP475-M^254^) decreased MbovP475’s ability to bind both promoters; however, the mutations N265A and Q266A (rMbovP475-^265^NQ^266^) did not change the binding ability of MbovP475 (Figure 7(c) and 7D and Fig. S9).

Complemented strains to the above mutants were constructed: CT9.55_W^246^-A, CT9.55_^242^NI^243^-AA, CT9.55_^242^NI^243^-NA, CT9.55_^242^NI^243^-NS, and CT9.55_^242^NI^243^-NG (Table S2). First, the expression of mutated MbovP475 in T9.55 was confirmed to be deficient, whereas normal expression was found in HB0801, CT9.55, and CT9.55_^242^NI^243^-NA, and only weak expression was observed in CT9.55_^242^NI^243^-AA, CT9.55_^242^NI^243^-NS, CT9.55_^242^NI^243^-NG, and CT9.55_W^246^-A (Figure 7(e)). Then the expression of CRYAB and MCF2L2 in BoMac cells infected with HB0801, T9.55, CT9.55, CT9.55_^242^NI^243^AA, CT9.55_W^246^, and CT9.55_^242^NI^243^-NA was compared. HB0801 and CT9.55 strains with normal expression of MbovP475 significantly suppressed the transcription of CRYAB and MCF2L2, whereas *M. bovis* T9.55 without MbovP475 expression and CT9.55_^242^NI^243^-AA with weak expression of MbovP475 did not suppress the transcription of both genes. The weak expression of MbovP475 in CT9.55_W^246^-A retained a low ability to suppress CRYAB transcription but lost this ability to suppress MCF2L2 transcription. Most importantly the mutation of I243A in MbovP475_^242^NI^243^-NA didn’t affect MbovP475 expression, but resulted in a loss of inhibition of transcription of both CRYAB and MCF2L2. These findings indicated that this residue was critical to MbovP475 function (Figure 7(f,g)). In the TALE, NI paired with Ade or Cyt, while NA paired with Gua. Therefore, NI at aa 242 and 243 were essential for binding of MbovP475 to the promoter fragments of CRYAB and MCF2L2 and suppression of their transcription.

### MbovP475 decreased cell viability through CRYAB and MCF2L2

**Figure 8.**
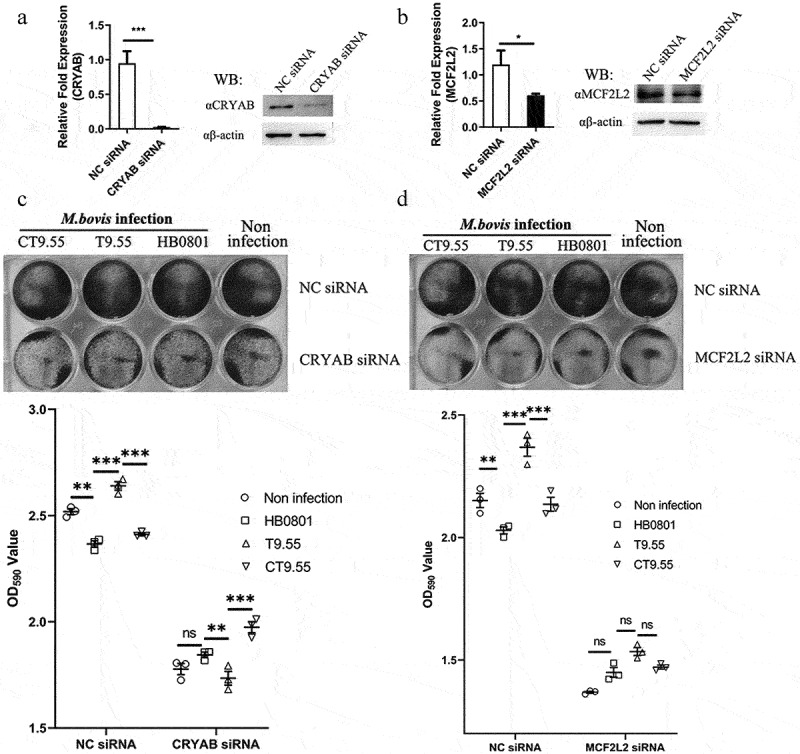
MbovP475 inhibition of cell viability by down regulation of MCF2L2 and CRYAB. (a, b) the expression of MCF2L2 and CRYAB in BoMac at 24 h post-transfection with siRNA was detected by qRT-PCR and western blotting assays. (c, d) the cell viability of cryab-knockdown or mcf2l2-knockdown BoMac cells infected with *M. bovis*. The cell viability of BoMac at 24 h post-infection with *M. bovis* (MOI = 5) was determined by crystal violet assay. One way ANOVA and Tukey’s test were used, *p <0.05, **p <0.01, ***p <0.001 indicate statistically significant differences. Data are representative one experiment of three independent biological replicates.

To further confirm that CRYAB and MCF2L2 are the targets of MbovP475, we constructed *cryab*-knockdown and *mcf2l2*-knockdown BoMac cell mutants by using corresponding siRNAs (Table S3). The expression deficiency of CRYAB and MCF2L2 at 24 h post-transfection was confirmed with RT-PCR and western blotting assay (Figure 8(a,b)). The *cryab*- and *mcf2l2*- knockdown BoMac mutants, as well as the wtBoMac transfected with NC siRNA, were then infected with HB0801, T9.55, and CT9.55, and crystal violet staining assay was used to detect cell viability. In the cells transfected with NC siRNA, significant differential viability among the four groups with or without MbovP475 expression was detected, thus indicating that MbovP475 decreased the cell viability. However, in cells transfected with CRYAB siRNA (Figure 8(c)), no difference in cell viability was observed, thus indicating that MbovP475 suppressed cell viability in these cells through CRYAB. Similarly, the cells transfected with MCF2L2 siRNA (Figure 8(d)) showed no significant difference among these four groups indicating the inhibitory effect of MbovP475 on cell viability was dependent on MCF2L2. Therefore, both CRYAB and MCF2L2 were the direct targets of MbovP475 in decreasing cell viability.

### MbovP475 affected the growth of intracellular mycoplasma

**Figure 9.**
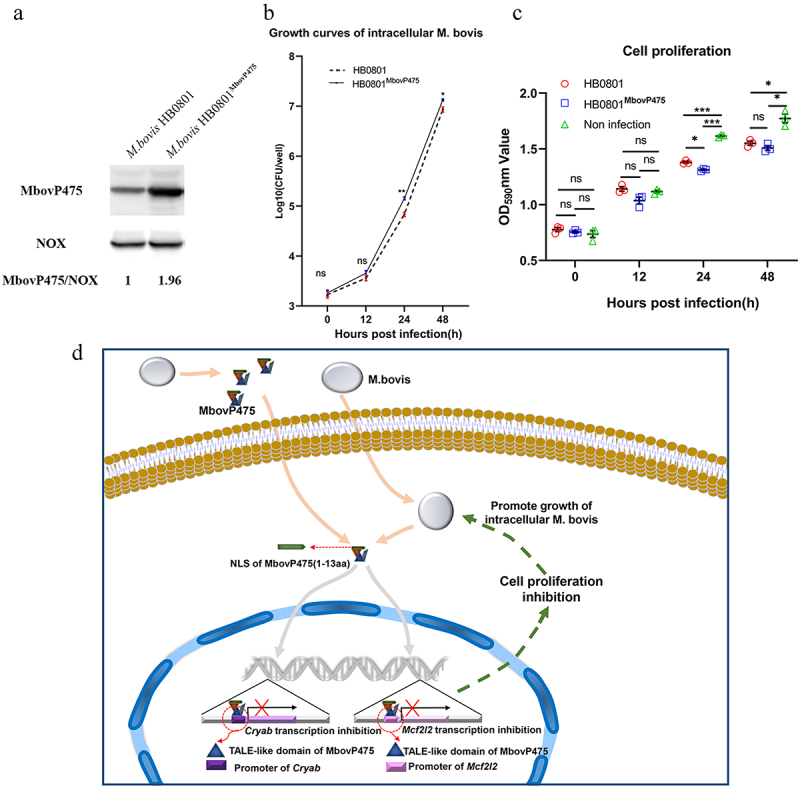
MbovP475 promoted the growth of intracellular mycoplasma. (a) the overexpression of MbovP475 in the *M. bovis* HB0801^MbovP475^ strains. The expression of MbovP475 in strains was detected by western blotting assay with the polyclonal antibodies against MbovP475. The NOX of *M. bovis* served as control. (b) the titres of intracellular *M. bovis*. The cells were infected with *M. bovis* and treated with gentamicin (400 μg/mL), and determined the CFU/well at 0, 12, 24, and 48 h post-infection. (c) Cell viability of BoMac infected with *M. bovis* HB0801^MbovP475^ or *M. bovis* HB0801. The cells were infected with *M. bovis* and treated with gentamicin (400 μg/mL), and wells were washed once with PBS and stained with 0.5% crystal violet solution at 0, 12, 24, and 48 h post-infection. Then, 1 mL of 95% ethanol was added to each well and incubated for 2 h after washing and air-drying. Then the optical density (OD) was measured at 590 nm. One way ANOVA and Tukey’s test were used, * *p* <0.05, **p <0.01, and ***p <0.001 and “ns” indicate statistically significant differences and no difference, respectively. Data are representative one experiment of three independent biological replicates. (d) Schematic diagram of the decrease in cell viability by MbovP475. The cell viability was decreased by the MbovP475 secreted by intracellular or extracellular *M. bovis* via inhibiting CRYAB and MCF2L2 transcription through binding the promoter fragment of these genes.

To assess whether MbovP475 affected the growth of *M. bovis* in infected cells, we used conventional gentamicin protection assay to detect the growth of intracellular mycoplasma. Since the MbovP475 knock-out mutant T9.55 was constructed using a transposon mutant library with pMT85 vector, which confers resistance to gentamicin, its complemented strains CT9.55 contains the gentamicin resistance gene either. Therefore, both T9.55 and CT9.55 strains were not suitable to Gentamicin protection assay. Alternately, the overexpression strain HB0801^MbovP475^ was correctly constructed for this assay. The western blotting assay verified that the expression of MbovP475 in this strain was 1.96 folds that in the wild-type strain (wtHB0801), while *M. bovis* NOX was used as the internal reference (Figure 9(a)). BoMac cells were incubated with either wtHB0801 or HB0801^MbovP475^ for 3 h, then switched to medium with gentamicin for 3 h that was taken as 0 h post-infection and further cultured with medium without gentamicin for 12, 24, and 48 h. The intracellular *M. bovis* was counted with conventional plating count assay. Immediately after gentamicin treatment (0 h), the number of intracellular *M. bovis* was similar, at approximately 10^3^ CFU/well for each strain; the colony numbers increased at 12 h, then displayed enhancement of growth, exceeding 2.1 folds, at 24 h post-treatment, with significant differences between the strains (*p* < 0.01). The colony numbers continued to rise at 48 h, reaching 10^7^ CFU/well, the significant difference between the strains was maintained (*p* < 0.05; Figure 9(b)). The above results indicated that MbovP475 promoted the intracellular *M. bovis* growth significantly at 24 h and 48 h post infection.

On the other hand, the cell viability was measured by crystal violet staining assay. Compared to the non-infection, both *M. bovis* HB0801 and *M. bovis* HB0801^MbvoP475^ decreased the cell viability at 24 h and 48 h post-infection (*p* < 0.01). HB0801^MbvoP475^ with over-expression of MbovP475 displayed a stronger inhibitory effect on BoMac cell viability than did HB0801 at 24 h post-infection (*p* < 0.05), as shown in Figure 9(c).

## Discussion

In the past 10 years, studies have increasingly revealed the ability of mammalian bacterial pathogens to directly attack cellular nuclei and regulate the expression of key genes involved in critical host responses, such as immunity, cell proliferation, or apoptosis [30]. An important mechanism involves secretion of nucleomodulins that directly attach to host cell DNA. However, the study of the secreted nucleomodulins of Mycoplasmas is just beginning. The only evidence for mycoplasmas previously reported has indicated that the secreted DNA methyltransferases of *M. hyorhinis* can enter host cell nuclei, where they methylate host genes and lead to cell tumour formation. The current research provides the first demonstration that *M. bovis* secreted TALEs-like nucleomodulin MbovP475, which targeted the promoters of CRYAB and MCF2L2 genes, and decreaseds cell viability.

### The NLS of MbovP475 is essential to its nuclear translocation

Our previous study revealed that MbovP475 is a secreted protein of *M. bovis* [27]. This study demonstrated that either the MbovP475 naturally secreted by *M. bovis* or purified recombinant rMbovP475 could be translocated from cellular cytoplasm into nuclei dependent on its NLS peptide (aa 1–13). However, the signal peptide (aa 1–21) of MbovP475 is overlapped with this NLS. Because MbovP475 without NLS could not enter cell nuclei, we think the lipoprotein MbovP475 should be secreted without cleavage of its signal peptide. As well known, the cleavage of signal peptides is a common way for mycoplasmas to secret their proteins. For example, the previous studies have revealed that the mycoplasma lipoprotein P80 of *M. hominis*, MALP-404 of *M. fermentans*, and MPN052 of *M. pneumoniae* were secreted with cleavage of their signal peptides [21,24,44]. But the alternative secretion ways might exist. Recently, some studies revealed that mycoplasmas including *M. bovis* might release metabolites, proteins, polysaccharides through extracellular vesicles (EVs) or an unknown secretion system [45,46]. Therefore, we speculated that MbovP475 of *M. bovis* might be secreted through EVs or other unknown routes rather than through cleavage of the signal peptide. The definitive mechanism through which MbovP475 is secreted remains to be investigated in the future.

### Tales-Like structure functions similarly in the secreted mycoplasma protein MbovP475

Although MbovP475 was demonstrated to decrease cell viability, both the prediction of typical TALEs structure and Chip-seq findings suggest this protein might execute multiple functions, which remains to be studied. TALEs family proteins are well understood DNA-binding proteins of plant pathogens. The TALEs proteins AvrBs3 family members mimic eukaryotic transcription factors, thereby modulating the expression of host genes [47]. These TALEs contain a repeat region consisting of several basic helix-loop-helix domains, and use the loop regions between two helices to bind DNA bases [48,49]. The current study revealed that MbovP475 has a leucine-rich repeat region (DUF285 domain) containing five helix-loop-helix domains with five predictive DNA-binding sites: NA at aa 211–212, NI at 242–243, NQ at 265–266, NQ at 290–291, and NQ at 315–316, located in the loop region. After comparison of the DNA binding ability of several MbovP475 mutants with southwestern blotting assay, we found that NI between aa 242 and aa 243 in the loop region were essential for MbovP475 binding to DNA. Because the conserved residues W246, M254, and F258 constructed the two-helix backbone, we speculated that the pair of NI with Ade or Cyt might determine the specificity of the DNA binding motif of MbovP475. This phenomenon was also supported by previous research on the genus *Xanthomonas* indicating that AvrBs3 mediated the specific interaction with DNA through 17.5 repeats in the central region [47]. Additional results confirmed that the mutation of both residues in the helix region significantly decreased their DNA-binding ability. Together, our findings indicated that MbovP475’s secondary structure may determine the DNA-binding ability, and the loop region between two-helix is critical to the binding specificity. Although MbovP475 uses the TALE repeat region to bind DNA similarly to other TALEs, the outcome might be different from those of other TALEs. For example, AvrBs3/PthA family effectors interfere with cellular activities through transcriptional activation of host genes [48]. The acidic transcriptional activation domain beside repeat region is essential for TALEs transcriptional activation of host gene [49]. Although this study demonstrated that MbovP475 binds the promoter sequences of CRYAB and MCF2L2 genes and inhibits their expression, as mentioned above, the full functions of MbovP475 remain to be explored in the future.

### MbovP475 decreased the cell viability of host cells

To fight hostile environment factors for their own survival is a universal rule for both pathogens and host cells. Nucleomodulins such as AnkA, AvrBs3, and PtpA interact with host DNA, thereby regulating the expression of defence genes [49–51]. Other nucleomodulins, such as NUE and RomA, directly target and modify nuclear histones, thereby regulating host gene expression [52,53], or interact with other regulatory nuclear proteins, thereby antagonizing the host immune response [54,55]. In this study, MbovP475 was shown to decrease host cell viability detected by Cell Counting Kit 8 (CCK-8) assay and crystal violet staining assay. The EdU assay and flow cytometry assay were used to reveal the mechanisms why cell viability was decreased. The results indicated MbovP475 arrested the cells at S stage during the cell cycle rather than induced apoptosis. Whether pyroptosis or necroptosis is associated with the decrease in cell viability needs to be investigated in the future. Although *M. bovis* and other mycoplasma species were previously reported to decrease cell proliferation either [56–58], and Mycoplasmas might regulate cell growth or death by producing reactive oxygen species (ROS), secreting components, and activation of caspase-3 [59], only this study provides the first evidence that *M. bovis* utilizes its nucleomodulin (MbovP475) to suppress cell proliferation by directly binding to the promoter regions of MCF2L2 and CRYAB genes and down-regulating their expression. CRYAB can retain unfolded proteins, decrease apoptosis, regulate oxidative stress and cell cycle, and stabilize the cytoskeleton [60]. Overexpression of CRYAB inhibits the cell cycle and promotes cell proliferation [36]. These previous findings support our results indicating that MbovP475 down-regulated CRYAB expression and decreased cell viability through induction of cell cycle arrest at S stage. MCF2L2 is a Rho family guanine nucleotide exchange factor that contains signalling molecules responsible for Rho protein activity [38]. Rho GTPases are critical during cell cycle progression and mitosis [61], in agreement with our findings regarding MbovP475 function on cell cycle. However, we searched published literature to screened potential candidates from 194 genes and selected all the seven genes known to be associated with the cell proliferation. It would be possible that other target genes of MbovP475 contribute to the decrease of cell viability This limitation might potentially have resulted in other target genes associated with cell proliferation being missed.

Because the mutant T9.55 and its complemented strains CT9.55 contained a gentamicin resistance gene and could not be counted with Gentamicin protection assay, we took the alternative approach by overexpressing Mbovp475 in *M. bovis* HB0801^MbovP475^. However, we only got 1.96 folds higher expression of Mbovp475. Probably it would be the reason that the effect of overexpressed MbovP475 on reduction of cell viability and increase of intracellular *M. bovis* growth was not as large as we expected although the change was already statistically different.

In conclusion, this study first identified a secreted TALEs-like nucleomodulin, MbovP475 of *M. bovis*, and demonstrated that it binds to the promoters of CRYAB and MCF2L2 genes, and down-regulates their expression, thereby decreasing cell viability. This study offers a new breakthrough in the understanding of mycoplasma pathogenesis.

## Materials and methods

### Ethics statement

The protocol (HZAUMO-2018-027) for animal experiments to make antiserum was approved by the Committee on the Ethics of Animal Experiments at Huazhong Agricultural University (Wuhan, China) and all experiments were conducted in strict accordance with the Guide for the Care and Use of Laboratory Animals, Hubei Province, China.

### Growth of bacterial strains and cells

The *M. bovis* strain HB0801 (GenBank accession no. NC_018077.1) was isolated from diseased cattle in Hubei province (China) in 2008 [62]. Mycoplasma strains were grown in pleuropneumonia-like organism (PPLO) media (BD Company, MD, USA), as previously described [63]. The *M. bovis* mutants were grown in the same PPLO medium but supplemented with 100 μg/mL gentamycin or 10 μg/mL puromycin. An *E. coli* strain DH5α (TransGen, Beijing, China) was grown in Luria – Bertani broth (LB) with proper antibiotics when necessary.

The BoMac cell line was kindly provided by Judith R. Stabel from the Johne’s Disease Research Project at the United States Department of Agriculture in Ames, Iowa and grown as described previously [64]. The HEK293T cell line was purchased from the China Center for Type Culture Collection and cultured in high-glucose Dulbecco’s modified Eagle’s medium (HyClone) supplemented with 10% heat-inactivated FBS (Gibco).

### Prediction of secreted TALEs-like nucleomodulins in M. bovis

The tandem repeats in the helix-loop-helix domains characterized by TALEs were associated with DNA-binding function. So the BioAider V1.334 was applied to search tandem repeats in each secreted protein with NLS [65]. Further we used SeqNLS (http://mleg.cse.sc.edu/seqNLS) [66] to predict the NLS in the C-terminal and *N*-terminal regions of published secreted proteins of *M. bovis* by our laboratory [27,35].

### Construction of the complementary strain for Mbov_0475 mutant

The Mbov_0475 knockout mutant T9.55 (*M. bovis* ΔMbov_0475) was identified from the transposon-mediated *M. bovis* mutant library previously constructed in this laboratory [11]. The mutated site was at nucleotide (nt) 555 of the Mbov_0475 coding sequence (CDS) or nt 555,965 of the *M. bovis* HB0801 genome.

To construct a complementary strain CT9.55 for this mutant T9.55, the sequence of the *M. agalactiae P40* promoter followed by the intact Mbov_0475 CDS was synthesized at Beijing Tianyi Huiyuan Bioscience & Technology Inc. (Wuhan, China) and ligated into the pOH/P plasmid after digestion with restriction enzyme *Not* I to generate the recombinant plasmid pCT-CT9.55. The T9.55 compatible cells were then transfected with pCT-CT9.55 to generate the complementary strain CT9.55 with a previously described method [27]. Single colonies were selected with 10 μg/mL puromycin in the medium and confirmed with DNA sequencing. The T9.55 and CT9.55 strains were cultured in PPLO medium (Becton, Dickinson and Company, MD, USA) containing 100 μg/mL gentamycin and 10 μg/mL puromycin, respectively, and their growth curves were determined in parallel with a standard plate counting method. A Q5® site-directed mutagenesis Kit (NEB, Beijing, China) was used to construct pCT-CT9.55_^242^NI^243^-AA, pCT-CT9.55_W^246^-A, pCT-CT9.55_^242^NI^243^-NA, pCT-CT9.55_^242^NI^243^-NS, and pCT-CT9.55_^242^NI^243^-NG with primers listed in Table S3, as described in the manufacturer’s manual. The *M. bovis* CT9.55_^242^NI^243^-AA where aa residues 242 to 243 (NI) were changed to alanine, CT9.55_W^246^-A where the residue 246 (W) was changed to alanine, CT9.55_^242^NI^243^-NA where the residue 243 (I) was changed to alanine, CT9.55_^242^NI^243^-NS where the residue 243 (I) was changed to serine and CT9.55_^242^NI^243^-NG where the residue 243 (I) was changed to glycine, were constructed using the above method.

The MbovP475 expression in mutant T9.55 and its complementary strain CT9.55 was evaluated with western blotting assay. Both strains were cultured in 20 mL PPLO medium with the necessary antibiotics for 36 h and precipitated by centrifugation at 12,000 × *g* for 10 min. The pellet of each strain was then suspended in 1 mL of PBS and lysed by sonication at 200 W on ice for 5 min. The proteins in the lysate were then separated with 10% SDS-PAGE and transferred onto a PVDF membrane (Millipore, Darmstadt, Germany). The membrane was incubated with mouse antiserum (1:500) directed against rMbovP475 described as below or rMbovP579 previously made by this lab [27,67] at room temperature for 1 h. After the membrane was washed, it was overlaid with the horseradish peroxidase (HRP)-conjugated goat anti-mouse IgG antibody (1: 5,000; Southern Biotech) for 1 h at room temperature. The bands on the membrane were then visualized with the WesternBright™ ECL western blotting detection kit (Advansta, CA, USA).

### Cloning, development of various constructs and expression

The genes encoding predicted TALEs-like effectors such as Mbov_0475 and Mbov_0280, a known secreted but not nuclear located protein as the negative control, were optimized based on the codon bias in mammal cells mainly by replacing tryptophan codon UGG in *M. bovis* with UGA in *E. coli* and synthesized by Beijing Tianyi Huiyuan Bioscience & Technology Inc. (Wuhan, China) and ligated into the pEGFP-C1 vector (Novagen, Darmstadt, Germany) by *Bam*H I and *Xho* I restriction endonucleases to obtain the recombinant plasmids pEGFP-475 and pEGFP-280, respectively. After validation by sequencing, these plasmids were transformed into *E. coli* DH5α for amplification (TransGen, Beijing, China). The endotoxin-free plasmids were prepared with Endo-free Plasmid Mini Kit II (Omega) and then stored at −20 °C until use.

Based on the predicted NLS of MbovP475, which includes two fragments of amino acid (aa), 1–13 and 28–38, Mbov_475 gene truncated sequences in the forms of 475^−/+^ by deleting aa 1–13, while keeping aa 28–38, 475^+/-^ by keeping aa 1 to 13, but deleting aa 28–38, and 475^−/−^ by deleting both aa 1–13 and aa 28–38, were commercially synthesized after optimizing the sequences based on *E. coli* codon bias and ligated into the pEGFP-C1 vector (Novagen, Darmstadt, Germany) by double digestion with *Bam*H I and *Xho* I to develop the recombinant plasmids pEGFP-475^−/−^, pEGFP-475^−/+^ and pEGFP-475^+/-^. The plasmids pCAGGS-HA-MbovP475 and pCAGGS-HA-MbovP475^Δ1-^^13^ were constructed at the same time. These plasmids were confirmed to be correct by sequencing, transformed into *E. coli* DH5α (TransGen, Beijing, China) and the endotoxin-free plasmids were prepared with the protocol described as above.

The gene Mbov_0475 was codon-optimized, synthesized and cloned into pET-30a to get pET-30a-475. The DNA-binding residues of MbovP475 were predicted by DP-binding [68] and then the truncated mutants of MbovP475 were constructed. The gene fragments encoding MbovP475 with deletion of different fragments, including MbovP475^∆DB1^ by deleting aa 243–275, MbovP475^∆DB2^ by deleting aa 299–332 and MbovP475^∆DB3^ by deleting aa 339–368, were amplified using the primers listed in Table S3 and then cloned into pET-30a to get pET-30a-475^∆DB1^, pET-30a-475^∆DB2^ and pET-30a-475^∆DB3^ (Table S1), all were confirmed to be correct by sequencing.

The DNA-binding residues in the DB1 region of MbovP475 were predicted by two methods. The proteins homologous to MbovP475 were identified with MolliGen 3.0 (http://services.cbib.u-bordeaux.fr/molligen/) [69]. The conserved amino acid residues within DB1 region of MbovP475 were analysed and mutated including MbovP475_W^264^ (W264A), MbovP475_M^254^ (M254A), and MbovP475_F^258^ (F258A). The mutated gene sequences were amplified using the primers listed in Table S3 and cloned into the pET30a to get pET30a-475-W^264^, pET30a-475-M^254^, and pET30a-475-F^258^, confirmed to be correct by sequencing. Referring to the crystal structure of a TALE PthXo1 binding to DNA [43], the secondary structure was predicted by Jpred 4 (http://www.compbio.dundee.ac.uk/jpred/). The plasmids with combined site mutations pET30a-475-^242^NI^243^, pET30a-475-^265^NQ^266^, and pET30a-475-^242^NI^243-265^NQ^266^ encoding MbovP475_^242^NI^243^ (N242A and I243A), MbovP475_^265^NQ^266^ (N265A and Q266A) and MbovP475_^242^NI^243^_^265^NQ^266^ were then commercially synthesized and confirmed to be correct by sequencing.

Next, *E. coli BL21* (TransGen, Beijing, China) was individually transformed with each of the constructs and the recombinant proteins were expressed after induction with 0.8 mM isopropyl β-d-1-thiogalactopyranoside (IPTG). The proteins were purified with nickel affinity chromatography (GE Healthcare, NJ, USA), as described previously [70].

## Antiserum development

The mouse antiserum directed against rMbovP579 or rNOX was previously prepared by our labmates [27,67,70]. The purified rabbit polyclonal antibody against MCF2l2 was produced by AtaGenix (Wuhan, China). Rabbit antiserum against rMbovP475 was prepared in this study from six-week-old New Zealand rabbits purchased from the Hubei Provincial Center for Disease Control and Prevention (Wuhan, China) and raised in the Animal Facility of Huazhong Agriculture University (Wuhan, China). Two rabbits were immunized by subcutaneous injection with 600 µg purified protein emulsified in an equal volume of Freund’s complete adjuvant (Sigma, USA) for the priming immunization or with Freund’s incomplete adjuvant for the subsequent boosters at an interval of 2 weeks. When the antiserum titres peaked, the rabbits were euthanized and bled. The antisera were collected, titrated and stored at −20°C for further use. The polyclonal antibody against MbovP475 was purified with AbCap A/G 4FF column to a concentration of 5 mg/mL (Smart Lifesciences, Jiangsu, China) as the manual described.

### Location of MbovP475 in BoMac cells

BoMac cells at a concentration of 1 × 10^5^ were seeded in each well of 12-well plates overnight. The cells in each well were transfected by 1 μg pEGFP-145, pEGFP-290, pEGFP-339, pEGFP-467, pEGFP-475, pEGFP-796, pEGFP-280, or pEGFP with 1.5 μL Lipofectamine 2000 (Invitrogen, Carlsbad, CA). Fluorescence microscopy was used to observe the localization of expressed proteins in the cells at 24 h post-transfection.

BoMac cells were also seeded at a density of 5 × 10^5^ cells per well in a six-well plate and incubated overnight at 37°C. The cells infected with either *M. bovis* HB0801 at 2.5 × 10^8^ CFU/well or incubated with purified rMbovP475 at 10 μg/well for 3, 6 and 12 h. The cytoplasmic and nuclear proteins of infected cells were extracted by a Minute^TM^ Cytoplasmic and Nuclear Fractionation Kit (Invent, Beijing, China) as the manual described. The cytoplasmic and nucleus proteins were separately resolved by SDS-PAGE and then transferred to polyvinylidene difluoride (PVDF) membranes (Millipore, Darmstadt, Germany). Immunodetection was achieved with antibodies against MbovP475, NOX, α-tubulin (abcam, MA, USA), or PARP (abcam, MA, USA). NOX, α-tubulin, and PARP served as the markers for *M. bovis*, cytosolic and nuclear fractions, respectively. The proteins on the PVDF membrane were visualized with a WesternBright^TM^ ECL Western blotting detection kit (Advansta, CA, USA).

The Mbov_0475 mutant T9.55 and its complement CT9.55 were used to infect BoMac described above. After 12 h, the cytoplasmic and nuclear proteins of infected cells were extracted and the localization of MbovP475 was detected by western blotting assay as mentioned above.

### Identification of the nuclear localization signal

To verify the NLS, the constructed plasmids were transfected into BoMac and the localization of expressed MbovP475 or truncated MbovP475 was observed by confocal microscopy. Then 1 × 10^5^ BoMac cells were propagated on a microscope coverslip in each well of 12-well plates overnight. The cells were transfected with 1.5 μg pEGFP-475, pEGFP-475^+/-^, pEGFP-475^−/+^, pEGFP-475^−/−^, or pEGFP with 1.5 μL Lipofectamine 2000 (Invitrogen, Carlsbad, CA) in each well. After 24 h, the cells were washed three times with PBS and fixed with 4% neutralized paraformaldehyde in PBS for 10 min, and permeabilized with 0.5% TritonX-100 for 5 min at room temperature. The cell nuclei were counter-labelled with 5 mg/mL DAPI (Beyotime, Shanghai, China). The slides were then cover-slipped and observed by confocal laser fluorescent microscopy (Olympus FV1000 and I×81, Tokyo, Japan). Then 1 × 10^5^ HEK293T cells were propagated on a microscope coverslip in each well of a 12-well plate overnight. The above method was used to confirm the NLS of MbovP475.

## Assays on cell viability, proliferation, and apoptosis

The viability of BoMac cells infected with *M. bovis* strains HB0801, T9.55, and CT9.55 was detected by CCK-8 assay (DOJINDO Laboratories, Kumamoto, Japan). The cells were seeded at a density of 5000 cells per well in 96-well plates and incubated overnight at 37 °C. They were then infected with the HB0801, T9.55, or CT9.55 strains in triplicate at an MOI of 5 for 12 h, 24 h, and 36 h, while the cells treated with PBS served as the negative control. Then each well was added 10 μL CCK-8 and incubated for 2 h. The absorbance at 450 nm was measured and the relative cell viability was calculated as follows:

$$\begin{array}{rl} & Relative\;cell\;viability\left(\%\right)=\left(OD_{sample}-OD_{blank}\right)\\ & \;\;\;\;\;\;\;\;\;\;\;\;\;\;\;\;\;\;\;\;\;\;\;\;\;\;\;\;\;\;\;\;\;\;\;\;\;\;\;\;\;\;\;\;\;\;\;\;\;/\left(OD_{NC}-OD_{blank}\right)\times 100\% \end{array}$$

The proliferation of BoMac cells was assessed with the EdU assay as described below. 1 × 10^5^ BoMac cells were seeded in each well of a 12-well plate overnight. The cells were respectively treated with 1 μM rMbovP475, rMbovP475^ΔDB1^, or rMbovP475^ΔDB2^ for 24 h or infected with the strains HB0801, T9.55, and CT9.55 at an MOI of 5 for 24 h, while the cells treated with PBS served as the blank. Before detection, 10 μM of EdU was added to the medium and incubated for 2 h. The cells were fixed with 4% paraformaldehyde and stained with the BeyoClick^TM^ EdU Cell Proliferation Kit (Beyotime, Shanghai, China) with Alexa Fluor 488 and Hoechst 33,342 as described by the manual. The stained cells were observed by fluorescence microscopy and quantified using Image-Pro plus 6.0 software.

BoMac cell cycles were detected with flow cytometry, with the cells infected as described above and then digested with 0.25% trypsin solution and fixed with ice-cold 70% ethanol at 4 °C overnight. The cells were stained with 50 μg/mL propidium iodide (PI) (KeyGEN BioTECH, Nanjing, China) and the DNA content of cells in each group was determined by flow cytometry (FACSVerse, Becton Dickinson, NJ, USA). All assays were repeated three times.

The apoptosis of BoMac treated with rMbovP475 was detected by Flow cytometry where 5 × 10^5^ BoMac cells were seeded into each well of a six-well plate and incubated overnight and treated with 1 μM rMbovP475 for 24 h. The cells were harvested and stained with an Annexin V-FITC Apoptosis Detection Kit (Vazyme, Nanjing, China) according to the manufacturer’s instruction. An Apoptosis Inducer Kit (Beyotime, Shanghai, China) was used as the positive control. The apoptotic cells were detected with Flow cytometry and the data analysed with the FlowJo VX software. The experiments were performed independently three times.

### Chromatin immunoprecipitation (ChIP) analysis

The BoMac cells were cultured in 10 cm dishes and infected with *M. bovis* HB0801 at an MOI of 500 for 12 h. ChIP analysis was performed using EZ-Magna ChIP^TM^ A/G kit (EMD Millipore Corporation, Billerica, MA, USA). The infected cells were crosslinked with 1% formaldehyde at room temperature for 10 min. Glycine was added for 5 min at a final concentration of 0.125 M. The cells were scrapped, pelleted, and resuspended in 0.5 mL Cell Lysis Buffer containing 1× Protease Inhibitor Cocktail II and re-precipitated. The resultant pellets were resuspended in Nuclear Lysis Buffer and sheared by sonication. The sheared chromatin in the supernatant was incubated with 10 µg purified rabbit polyclonal IgG againstMbovP475 and 20 μL fully suspended protein A/G magnetic beads. The magnetic separator was used to pellet the Ig-bound protein A/G magnetic beads after incubation overnight at 4 °C with rotation. The bead pellet was washed sequentially by resuspending the beads in 0.5 mL Low Salt Wash Buffer, High Salt Wash Buffer, LiCl Wash Buffer, and TE Buffer in order. The Protein A/G bead-IgG/chromatin complex was pelleted by magnetic separator, the supernatant was removed carefully and DNA was eluted from the beads with Proteinase K at 62 °C with shaking and extracted using Spin Filters in the Kit. The DNA samples were subject to be sequenced by Novogene (Tianjin, China). The target genes with an enrichment of >1.5-folds and corrected *p*-values <0.05 in comparison with the total input DNA before enrichment with anti-MbovP475 antibody were selected to be further analysed by KEGG pathway enrichment analysis using the KABOS online tool (http://kobas.cbi.pku.edu.cn/kobas3/genelist/).

### Quantitative real-time polymerase chain reaction (qPCR) and western blotting assay

BoMac cells were seeded at a density of 5 × 10^5^ cells per well in six-well plates and infected with *M. bovis* strains at an MOI of either 5 or 500 then incubated overnight at 37 °C for 12 h. The cells in each well were lysed with 1 mL TRIzol® Reagent (life technologies, Carlsbad, CA, USA). Total RNA was extracted and reverse-transcribed into cDNA by an HiScript Q RT SuperMix for qPCR (Vazyme, Nanjing, China). The RT-PCR was performed using an Applied Biosystems Viia 7 Fast Real-Time qPCR System (Applied Biosystems, Carlsbad, CA, USA) and SYBR Green Master Mix (Vazyme, Nanjing, China). Each sample was in triplicate and each experiment repeated independently three times. Data were analysed by the 2^−∆∆CT^ method and normalized to the expression of β-actin. The primers used in this step were listed in Table S3.

BoMac cells were seeded in a six-well plate and infected with *M. bovis* as described above. The cells were treated with 1 μM rMbovP475, rMbovP475^ΔDB1^, and rMbovP475^ΔDB2^ for 24 h. The cells in each well were lysed with 250 μL RAPI buffer (Sigma). Then the whole cell proteins were resolved with SDS-PAGE and transferred onto polyvinylidene difluoride (PVDF) membranes (Millipore, Darmstadt, Germany). The proteins were immunodetected with antibodies against CRYAB (Abcam, ON, Canada), MCF2L2, and β-actin as the internal reference. The membranes were then incubated with HRP-conjugated goat anti-mouse IgG antibody or HRP-conjugated goat anti-rabbit IgG antibody (1:5,000) (Southern Biotech) for 1 h at room temperature. The bands on the membrane were visualized with Western BrightTM ECL (Advansta).

### Identification of interactive DNA of MbovP475 with EMSA and SPR assays

EMSA and SPR were performed to identify the DNA interacting components of MbovP475. First, the biotin labelled promoter fragments of CRYAB (+108 bp to + 167 bp), CRYAB (−120 bp to −61 bp), and MCF2L2 (+53 bp to + 112 bp) were synthesized by TSINGKE Biological Technology (Wuhan, China). The EMSA was performed following the manual of LightShift® Chemiluminescent EMSA Kit (Thermo Scientific, Rockford, IL, USA), by incubating 5 μg of rMbovP475 with 50 fmol biotin labelled CRYAB promoter fragments (+108 bp to + 167 bp), CRYAB promoter fragments (−120 bp to −61 bp), or MCF2L2 promoter fragments (+53 bp to + 112 bp) in the binding buffer for 20 min at room temperature. Because the binding site of MbovP475 in CRYAB promoter fragments is around +138 bp, the CRYAB promoter fragments (−120 bp to −61 bp) served as negative control. The reaction mixtures were resolved with 5% non-denaturing polyacrylamide gel and transferred to nylon membranes (Beyotime, Shanghai, China). The DNA oligomers were UV-light crosslinked to the membrane and the labelled probes were detected by Chemiluminescence.

SPR assay was used to determine the affinity and kinetics of interaction between rMbovP475 and promoter fragments. The rMbovP475 was captured on a charged NTA sensor chip via C-terminal His-tag, while the two fragments at five concentrations (0.5 nM, 1 nM, 2 nM, 4 nM, and 8 nM). The binding between the rMbovP475 and these promoter fragments was detected, and the affinity calculated by Biacore T2000 (GE Healthcare Biosciences, MA, USA).

### Luciferase reporter assays

HEK293T cells were seeded at 2.5 × 10^4^ cells per well in a 12-well plate and co-transfected with 1 μg pGL3-basic-promoter vector or mock vector and vector encoding HA-MbovP475 or 1 μg HA-MbovP475^Δ1-^^13^. The amplification of 700 bp from the TSS as the promoters for this assay was conducted by using the primers listed in Table S3 and pRL-TK (100 ng) was used as an internal control. The cells were harvested and analysed for firefly luciferase and renilla luciferase activity using the dual luciferase reporter assay kit (Promega, MI, USA).

### Southwestern blotting assay on interaction between DNA and MbovP475

The Southwestern blotting assay was performed as described previously with some modification [71]. The wild-type protein rMbovP475 or its mutants in the predicted DNA binding domains were mixed with sample buffer of 30 mM Tris-Cl at a pH of 6.8, 30% glycerol (vol/vol), 10% SDS (wt/vol), and 1 mM DTT, 0.002% (wt/vol) bromophenol blue and boiled for 5 min. Then, 2 μg each of proteins or BSA were resolved with 10% denaturing polyacrylamide gel and transferred to a nitrocellulose membrane (Beyotime). The proteins were re-naturized by placing the membrane in a small transparent plastic box with 5 mL TNED buffer of 10 mM Tris at pH of 7.5, 50 mM NaCl, 0.1 mM EDTA and 1 mM DTT containing 5% skim milk and incubating overnight at room temperature with shaking at 50 rpm. The membrane was then incubated with 3 mL TNED buffer containing 20 pmol biotin labelled promoter fragments at room temperature for 12 h. After washing membrane three times for 5 min each shaking at 50 rpm in 5 mL TBST, the membrane was incubated with 5 mL TBST buffer containing 17 μL stabilized Streptavidin-HRP conjugate at RT for 1 h. The proteins that specifically interacted with the promoter fragments were visualized with WesternBright™ ECL (Advansta, CA, USA) after three washes with TBST.

### Cell viability of cryab-knockdown or mcf2l2-knockdown BoMac cells

The small interfering RNAs (siRNAs) targeting *cryab* and *mcf2l2* genes and negative control (NC) siRNA with no specific target were synthesized by a commercial company (GenePharma, Suzhou, China) and listed in Table S3. The BoMac cells were seeded at a density of 1 × 10^5^ cells per well in 12-well plates and incubated overnight at 37 °C. Before transfection, each well contained 1 mL fresh RPMI 1640 medium containing 10% FBS. Then, 50 pmol of siRNA was diluted in 100 μL of jetPRIME® buffer and 5 μL of jetPRIME® reagent was added to the buffer. The mixture was added into the wells after incubating for 10 min. The expression of *cryab* and *mcf2l2* genes was detected at 24 h post-transfection by RT-PCR and western blotting assay described above.

To evaluate the cell viability of *cryab*-knockout and *mcf2l2*-knockout BoMac cells infected with *M. bovis* strains HB0801, T9.55, and CT9.55 at 24 h post-transfection at an MOI of 5 for 24 h, the cell number in each well were determined by staining cell monolayers with crystal violet. Briefly, the cells in 12-well plates were washed with PBS and then stained for 30 min with 0.5% crystal violet solution. The plates were air-dried after washing with water and then photographed. Subsequently, 1 mL of 95% ethanol was added to each well and incubated at room temperature for 2 h before the optical density was measured at 590 nm.

### Gentamicin protection assay on effect of MbovP475 on intracellular M. bovis growth

The *M. bovis* HB0801 was transfected with pCT-CT9.55 to generate the overexpression strain HB0801^MbovP475^. Single colonies were selected with puromycin (10 μg/mL) in the medium and then confirmed with DNA sequencing. The expression of MbovP475 was detected with western blotting assay as previously described.

The BoMac cells were seeded at a density of 2 × 10^5^ cells per well in 12-well plates and incubated overnight at 37°C. The cells were infected with *M. bovis* HB0801 or HB0801^MbovP475^ at an MOI of 5 for 3 h, then washed three times with PBS and suspended in fresh RPMI1640 medium supplemented with 400 µg/mL gentamicin. The plate was incubated for three more hours and washed three times with PBS. Fresh RPMI1640 medium containing 10% FBS without gentamicin was added to each well and further incubated. At 0,12, 24, and 48 h post-infection, the wells were washed three times with PBS and the bacterial number in CFU per well calculated using the previously described method [72]. All assays were performed in triplicates in two independent experiments.

### Effect of MbovP475 overexpression on cell viability

The BoMac cells were infected with either *M. bovis* HB0801 or HB0801^MbovP475^, and the cell viability was measured by crystal violet staining assay mentioned above at 0, 12, 24, and 48 h post-infection.

## Statistical analysis

Statistical analyses were performed in GraphPad Prism version 5 statistical software (GraphPad Software, La Jolla, CA, USA). The data were expressed as mean ± standard error of the mean (SEM). Two-tailed Student’s *t*-test was used for single comparison for the parametric data (viability, proliferation, apoptosis, bacterial counting, changed folds of gene expression, etc). One-way ANOVA and Post Hoc test (Tukey’s test) for equal population variation were used for multiple comparisons for the parametric data, while fold-change method and false discovery rate (FDR) approach were used for non-parametric data (genes enrichment). The *p* values <0.05 were defined as significantly difference, and *p* values <0.05, 0.01, 0.001 were marked respectively as *, **, and *** in the figures.

## Supplementary Material

Supplemental Material

## Data Availability

The ChIP-seq data have been deposited in the Gene Expression Omnibus (GEO) database under accession code GSE179651.
