# Supplementary material for: Novel mycoplasma nucleomodulin MbovP475 decreased cell viability by regulating expression of CRYAB and MCF2L2
Source: Virulence. 2022 Sep 19;13(1):1590–613. doi: 10.1080/21505594.2022.2117762 (PMC9487752; doi:10.1080/21505594.2022.2117762)
Supplement: Supplemental Material [file KVIR_A_2117762_SM4774.zip › supplementary/Supplementary Figures and Supplementary Table.docx]

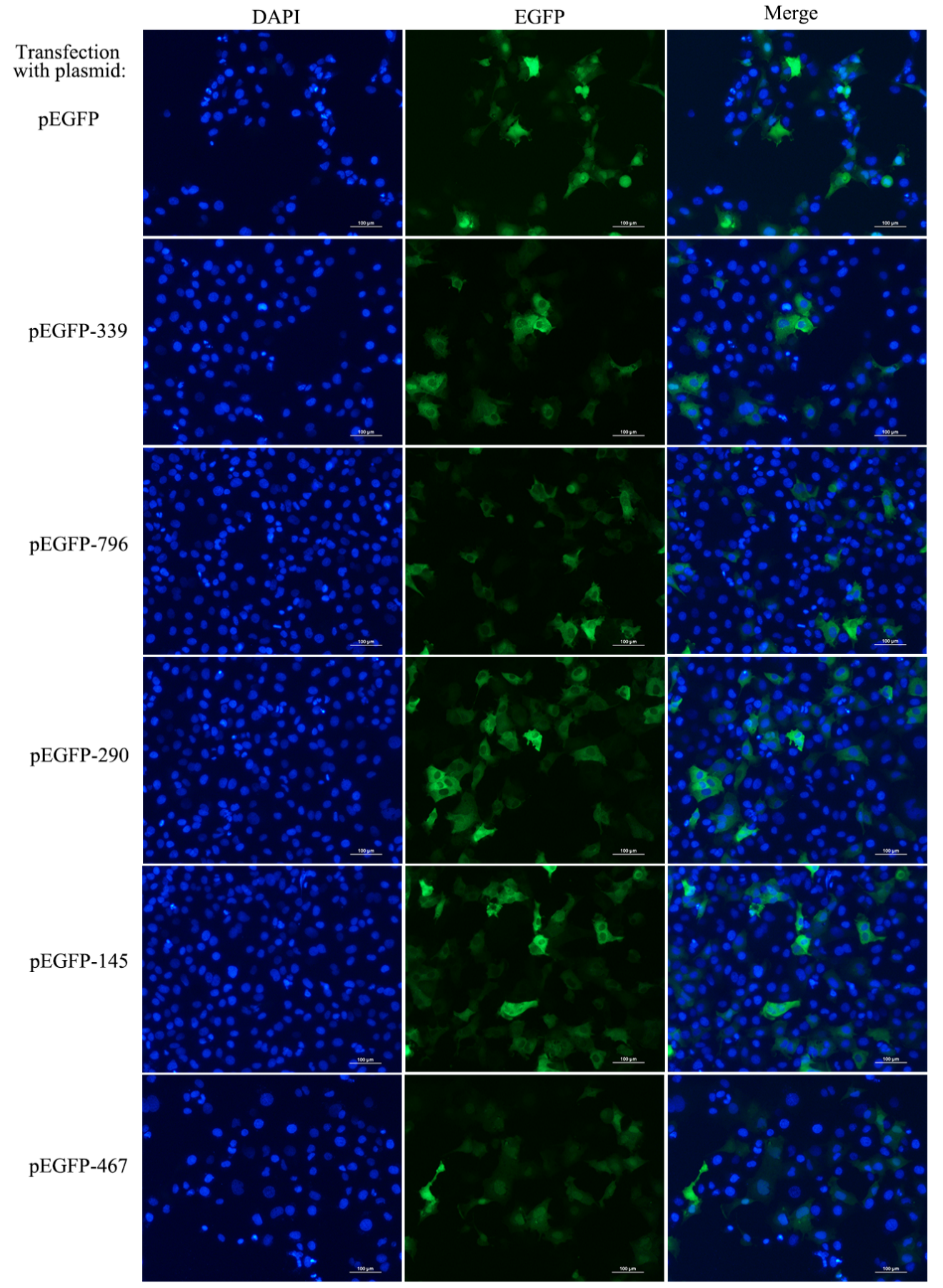


**Fig. S1** The nuclear localization of predicted secreted necleomodulins. The localization of MbovP145, MbovP290, MbovP339, MbovP467, or MbovP796 in BoMac was detected by fluorescence microscopy at 24 h post-transfection.


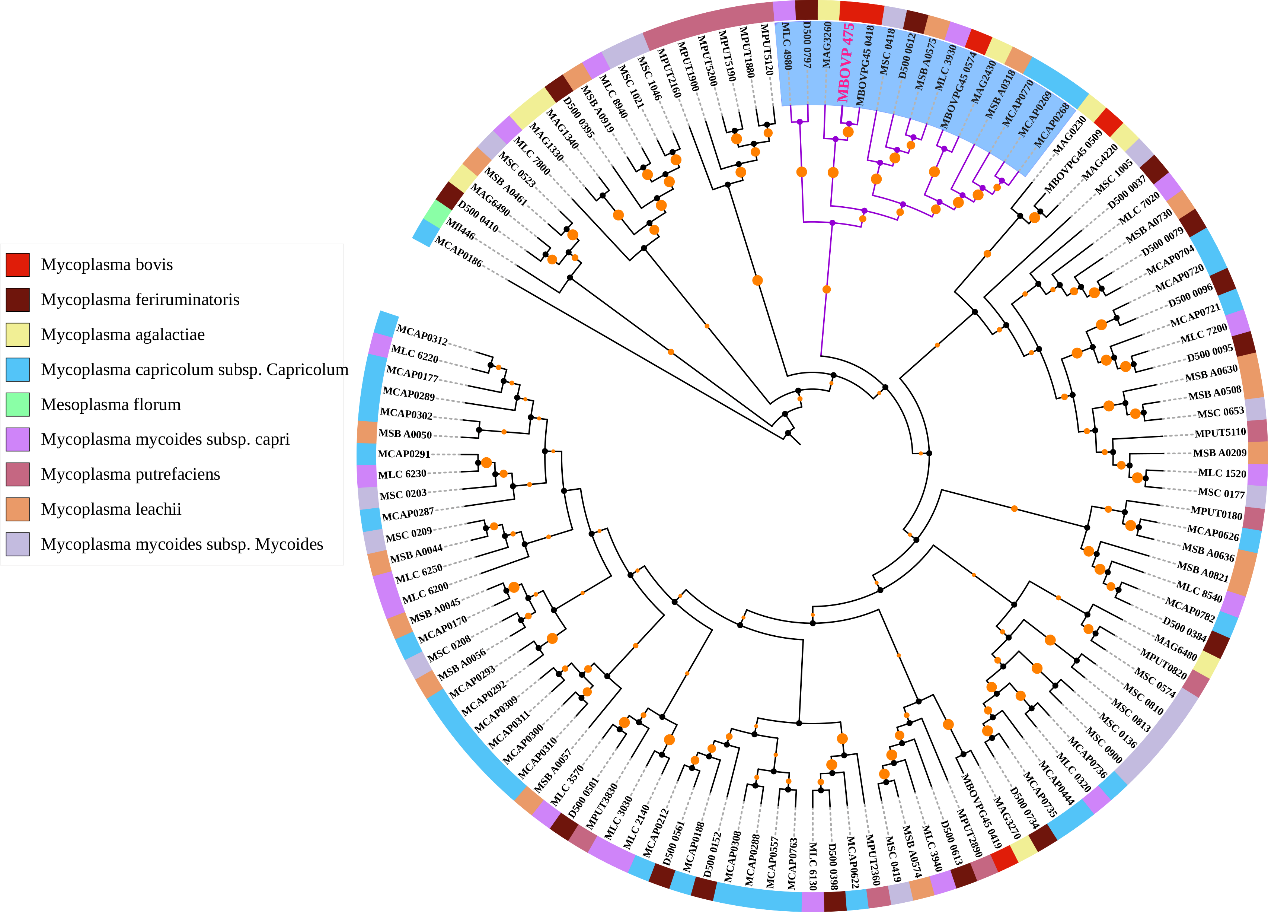


**Fig. S2** The proteins homologous to MbovP475. These proteins were identified with MolliGen 3.0. The phylogenetic trees were constructed using the neighbor-joining method with MEGA6 and the image was optimized with iTOL online service.


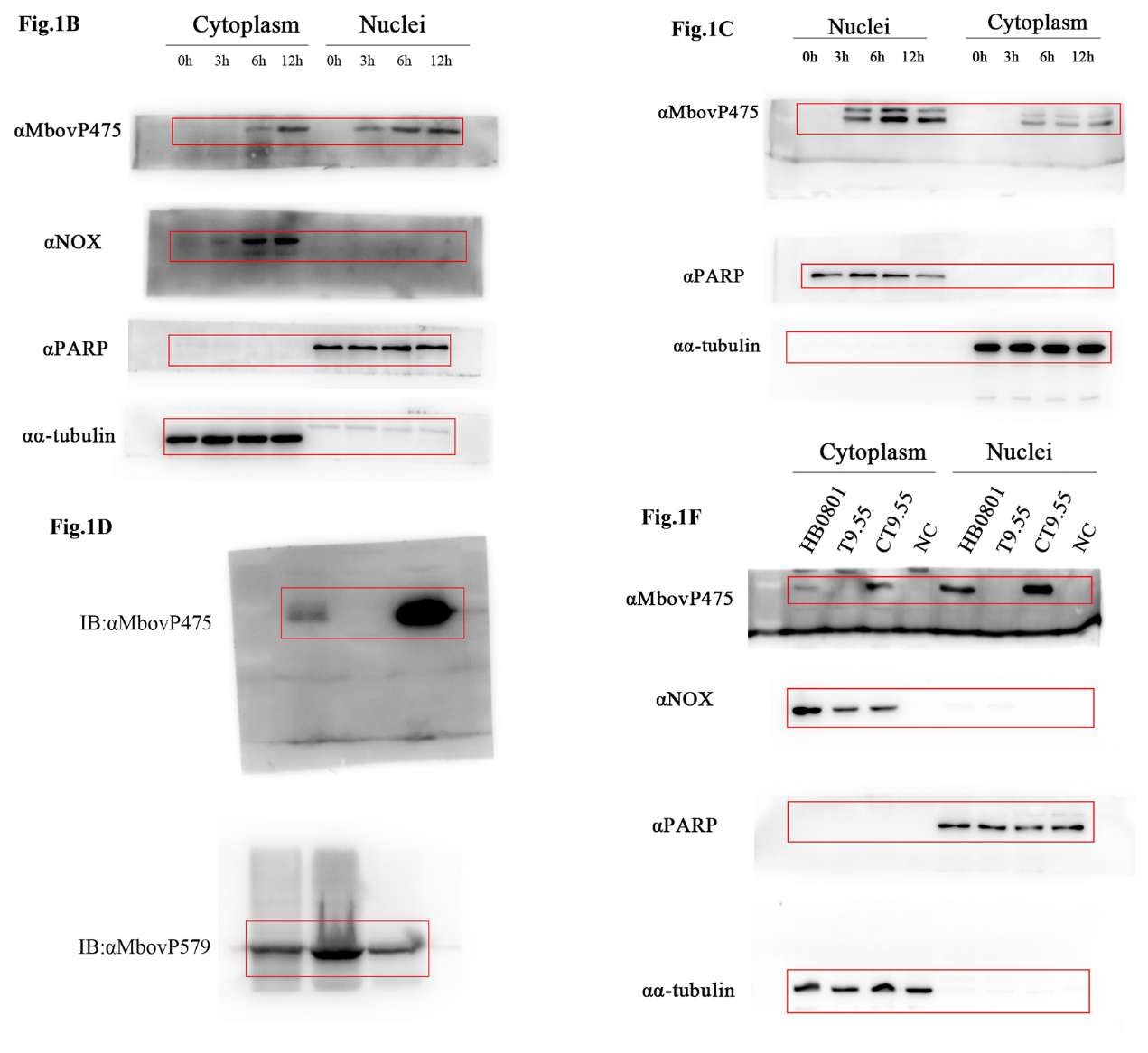


**Fig. S3** Original uncropped scans of blots for Figure 1.


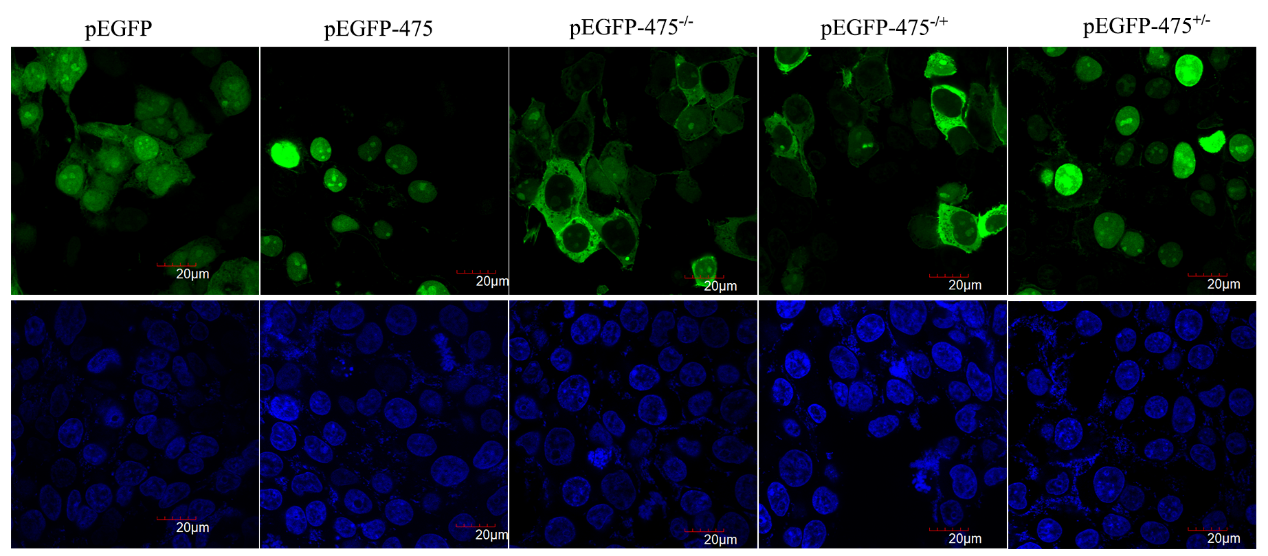


**Fig. S4** The pictures before merging for Fig.2C.


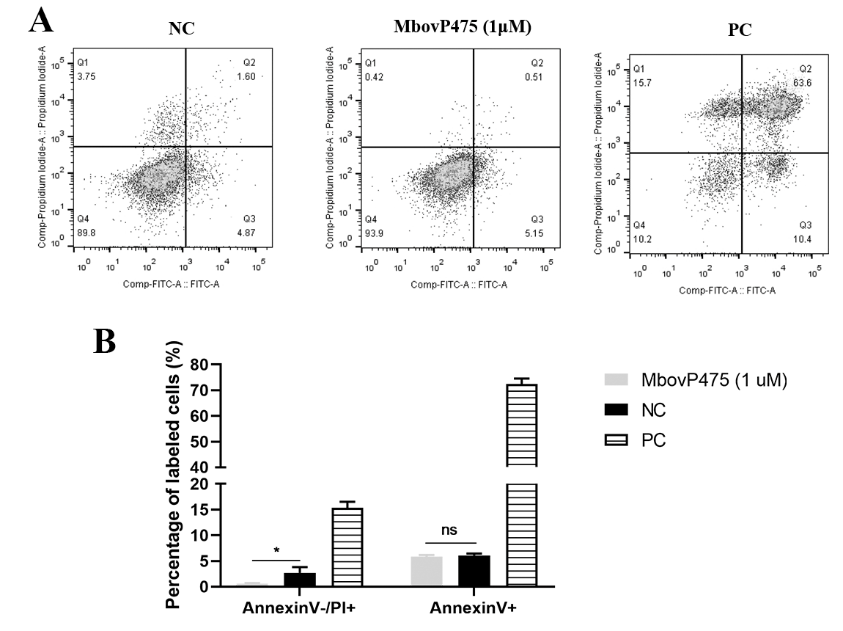


**Fig. S5** MbovP475 did not induce apoptosis of BoMac. (A,B) The apoptosis of BoMac was detected by flow cytometry. BoMac cells were treated with 1 μM rMbovP475 for 24 h, and the cells treated with PBS or apoptosis inducers served as negative control and positive control. The Annexin V-stained cells represented the apoptosis, and the cells only stained by PI represented necrosis. Two-tailed Student’s *t*-test, **p* < 0.05, ***p* < 0.01, ****p* < 0.001, and “ns” indicate statistically significant differences and no difference, respectively. Data are representative one experiment of three independent biological replicates.


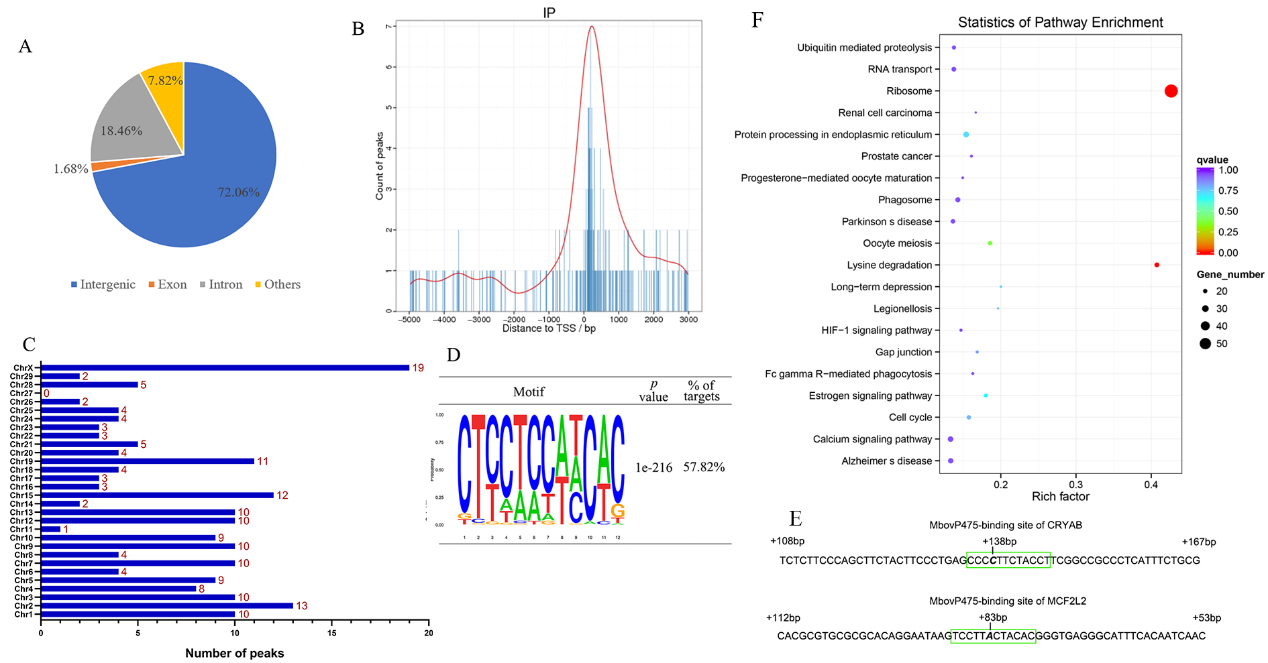


**Fig. S6** Genome-wide analysis of MbovP475-binding sites in BoMac. (A) Genomic distribution of potential MbovP475-binding sites in BoMac cells. (B) The numbers and position of MbovP475-binding sites within 2 kb around the near transcription start site (TSS). (C) Distribution of the chromosomal location of potential MbovP475-binding sites. (D) The most probably MbovP475 consensus motifs were identified by MEME motif analysis. The *p*-value was calculated using TOMTOM match statistics. “% of targets” means the percentage of this motif in the MbovP475-binding peaks. (E) Top 20 enriched KEGG pathways was analyzed by KABOS. (F) The MbovP475-binding fragment of CRYAB and MCF2L2. The bold italic base represents the position of the summit of MbovP475-binding peak located in genes form TSS. The green box represents the consensus motif.


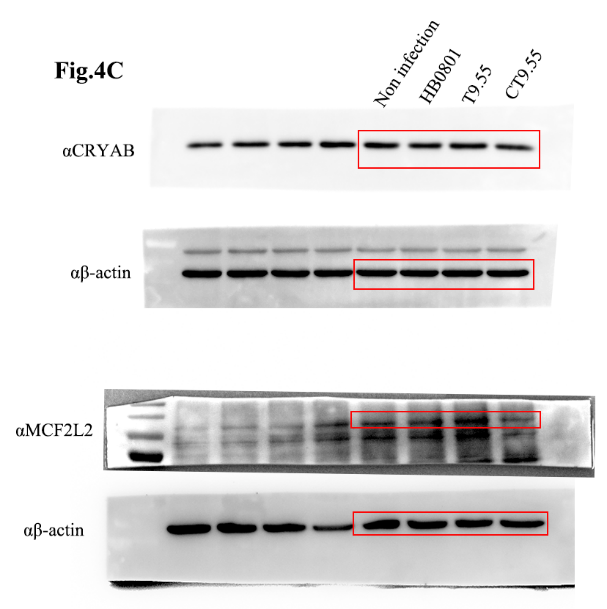


**Fig. S7** Original uncropped scams of blots for Figure 4.


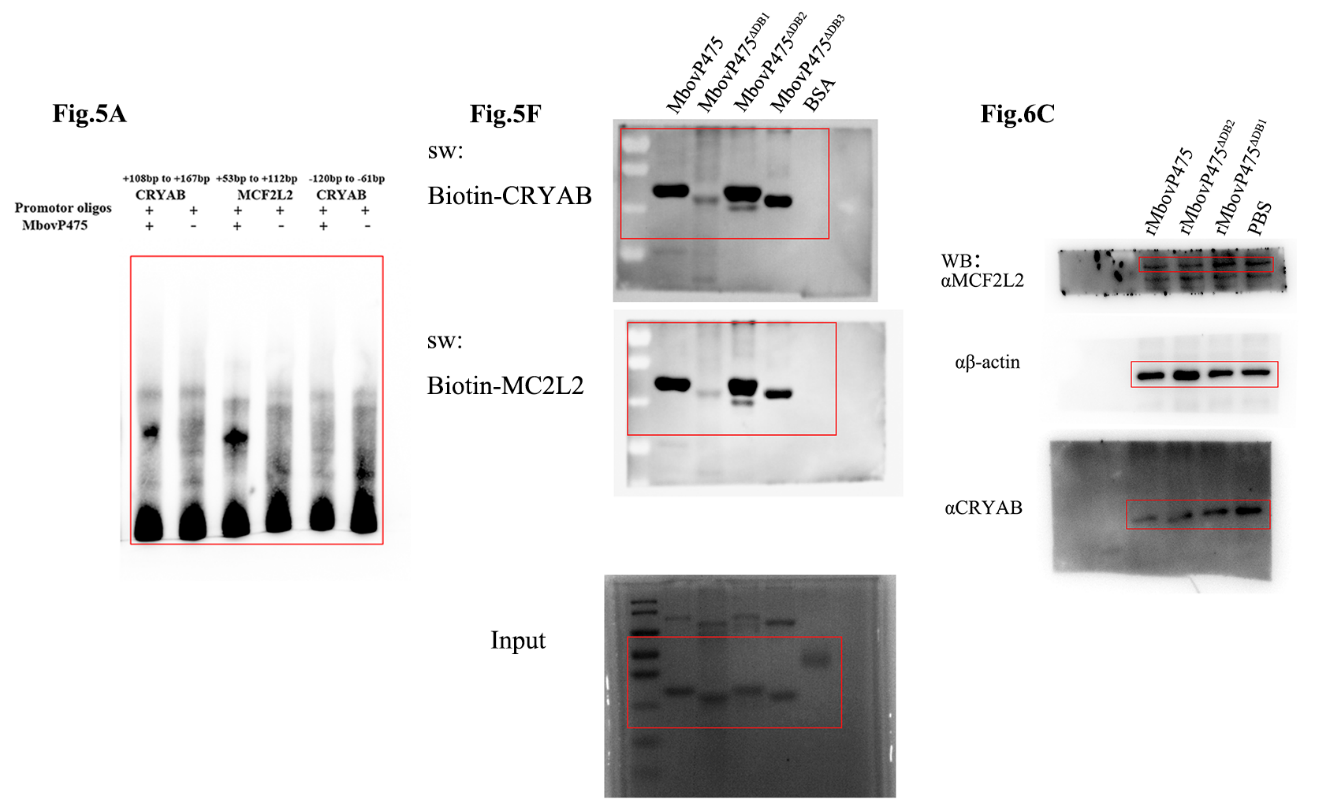


**Fig. S8** Original uncropped scams of blots for Figures 5 and 6.


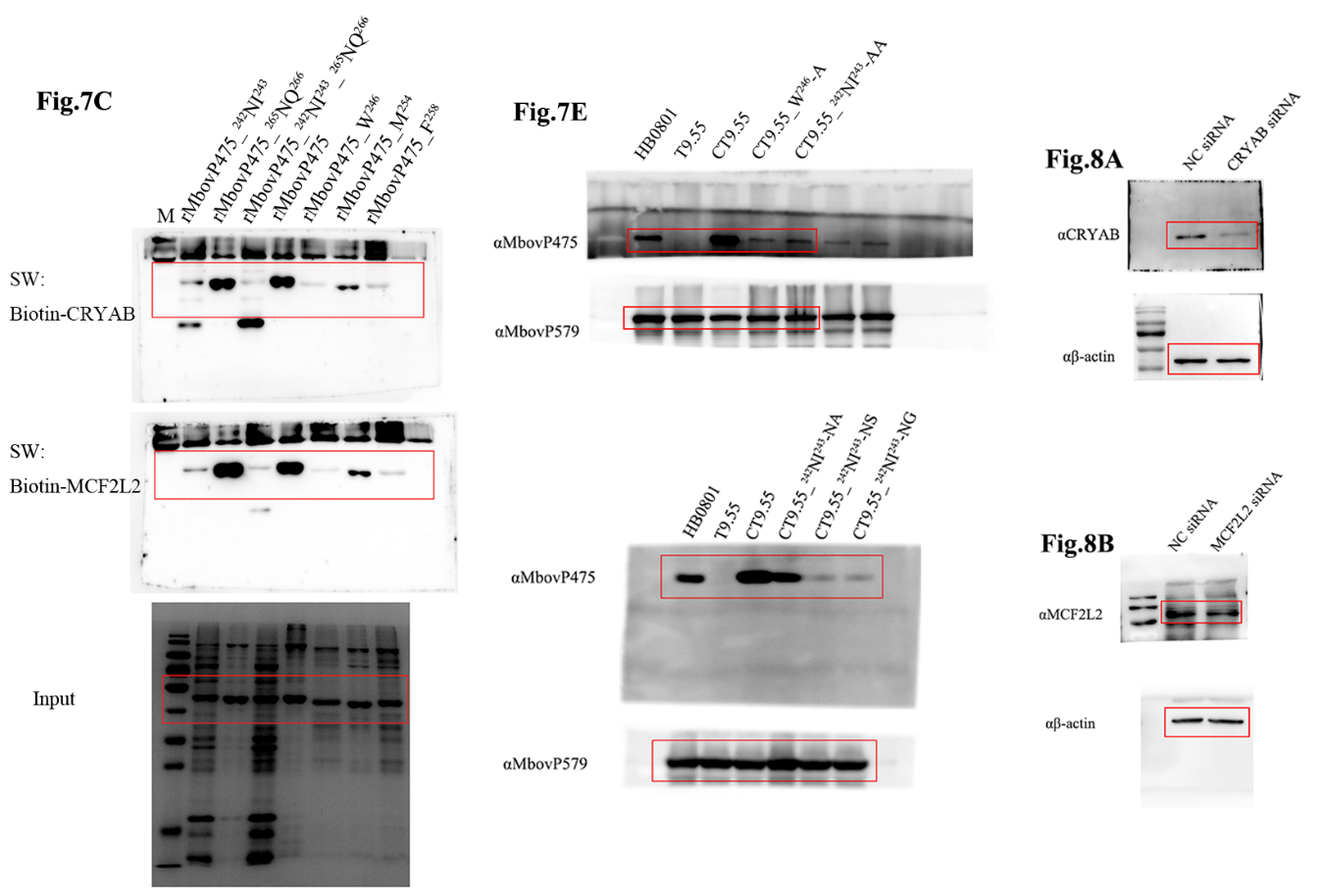


**Fig. S9** Original uncropped scams of blots for Figures 7, and 8.

**Table S1.** Plasmids used in the present study

| **Name** | **Description** |
| --- | --- |
| pOH/P | Complementation plasmid, Puro^r^ |
| pCT-CT9.55 | pOH/P plasmid inserted with Mbov_475 coding region under the control of the M. agalactiae P40 lipoprotein promoter, Puro^r^ |
| pCT-CT9.55_^242^NI^243^-AA | pOH/P plasmid inserted with mutant Mbov_475 coding region under the control of the M. agalactiae P40 lipoprotein promoter, encoding MbovP475_^242^NI^243^, in which amino acid residues 242 to 243 (NI) were changed to alanine, Puro^r^ |
| pCT-CT9.55_W^246^-A | pOH/P plasmid inserted with mutant Mbov_475 coding region under the control of the M. agalactiae P40 lipoprotein promoter, encoding MbovP475_W^246^, in which amino acid residues W246 was changed to alanine, Puro^r^ |
| pCT-CT9.55_^242^NI^243^-NA | pOH/P plasmid inserted with mutant Mbov_475 coding region under the control of the M. agalactiae P40 lipoprotein promoter, encoding MbovP475_^242^NI^243^-NA, in which amino acid residues I243 was changed to alanine, Puro^r^ |
| pCT-CT9.55_^242^NI^243^-NS | pOH/P plasmid inserted with mutant Mbov_475 coding region under the control of the M. agalactiae P40 lipoprotein promoter, encoding MbovP475_^242^NI^243^-NS, in which amino acid residues I243 was changed to serine, Puro^r^ |
| pCT-CT9.55_^242^NI^243^-NG | pOH/P plasmid inserted with mutant Mbov_475 coding region under the control of the M. agalactiae P40 lipoprotein promoter, encoding MbovP475_^242^NI^243^-NG, in which amino acid residues I243 was changed to glycine, Puro^r^ |
| pET-30a(+) | Expression plasmid, Kan^r^ |
| pEGFP-C1 | Expression plasmid, Amp^r^ |
| pEGFP-145 | pEGFP-C1 vector encoding MbovP145 with EGFP tag, Amp^r^ |
| pEGFP-280 | pEGFP-C1 vector encoding MbovP280 with EGFP tag, Amp^r^ |
| pEGFP-290 | pEGFP-C1 vector encoding MbovP290 with EGFP tag, Amp^r^ |
| pEGFP-339 | pEGFP-C1 vector encoding MbovP339 with EGFP tag, Amp^r^ |
| pEGFP-467 | pEGFP-C1 vector encoding MbovP467 with EGFP tag, Amp^r^ |
| pEGFP-475 | pEGFP-C1 vector encoding MbovP475 with EGFP tag, Amp^r^ |
| pEGFP-796 | pEGFP-C1 vector encoding MbovP796 with EGFP tag, Amp^r^ |
| pEGFP-475^+/-^ | pEGFP-C1 vector encoding truncated MbovP475 (missing 28-38aa) with EGFP tag, Amp^r^ |
| pEGFP-475^-/+^ | pEGFP-C1 vector encoding truncated MbovP475 (missing 1-13aa) with EGFP tag, Amp^r^ |
| pEGFP-475^-/-^ | pEGFP-C1 vector encoding truncated MbovP475 (missing 1-13aa and 28-38aa) with EGFP tag, Amp^r^ |
|  |  |
|  |  |
| pET30a-475 | pET-30a vector encoding recombinant protein rMbovP475, Kan^r^ |
| pET30a-475^∆DB1^ | pET-30a vector encoding recombinant protein rMbovP475^∆DB1^ (missing 243-275aa), Kan^r^ |
| pET30a-475^∆DB2^ | pET-30a vector encoding recombinant protein rMbovP475^∆DB2^ (missing 299-332aa), Kan^r^ |
| pET30a-475^∆DB3^ | pET-30a vector encoding recombinant protein rMbovP475^∆DB3^ (missing 339-368aa), Kan^r^ |
| pET30a-475-W^246^ | pET-30a vector encoding recombinant protein MbovP475_W^246^, in which amino acid residue W246 was changed to alanine, Kan^r^ |
| pET30a-475-M^254^ | pET-30a vector encoding recombinant protein MbovP475_M^254^, in which amino acid residue M254 was changed to alanine, Kan^r^ |
| pET30a-475-F^258^ | pET-30a vector encoding recombinant protein MbovP475_F^258^, in which amino acid residue F258 was changed to alanine, Kan^r^ |
| pET30a-475-^242^NI^243^ | pET-30a vector encoding recombinant protein MbovP475_^242^NI^243^, in which amino acid residues 242 to 243 (NI) were changed to alanine, Kan^r^ |
| pET30a-475-^265^NQ^266^ | pET-30a vector encoding recombinant protein MbovP475_^265^NQ^266^, in which amino acid residues 265 to 266 (NQ) were changed to alanine, Kan^r^ |
| pET30a-475-^242^NI^243^-^265^NQ^266^ | pET-30a vector encoding recombinant protein MbovP475_^242^NI^243^-^265^NQ^266^, in which amino acid residues 242 to 243 (NI) and 265 to 266 (NQ) were changed to alanine, Kan^r^ |
| promoter-pGL3-Basic (MCF2L2) | pGL3-Basic vector containing promoter region of MCF2L2 (-500 bp to +200 bp from the TSS) |
| promoter-pGL3-Basic (AMIGO1) | pGL3-Basic vector containing promoter region of AMIGO1 (-400 bp to +286 bp from the TSS) |
| promoter-pGL3-Basic (CRYAB) | pGL3-Basic vector containing promoter region of CRYAB (-500 bp to +201 bp from the TSS) |
| promoter-pGL3-Basic (RBM17) | pGL3-Basic vector containing promoter region of RBM17 (-500 bp to +205 bp from the TSS) |
| promoter-pGL3-Basic (HMGB1) | pGL3-Basic vector containing promoter region of HMGB1 (-490 bp to +225 bp from the TSS) |
| pCAGGS-HA | An empty plasmid encoded HA tag in cells after transfection, Amp^r^ |
| pCAGGS-HA-475 | pCAGGS-HA vector encoded MbovP475 in cells after transfection, Amp^r^ |
| pCAGGS-HA-475^∆1-13^ | pCAGGS-HA vector encoded truncated MbovP475 (missing 1-13 aa) in cells after transfection, Amp^r^ |

**Table S2.** Strains and used in the present study

| **Name** | **Description** | **Reference** |
| --- | --- | --- |
| HB0801 | Mycoplasma bovis wild type strains | [63] |
| T9.55 | MbovP475 knock-out strains constructed from HB0801 | [11] |
| CT9.55 | T9.55 transformed with plasmid pCT-CT9.55, which encode wild type MbovP475 | Constructed in this study |
| HB0801^MbovP475^ | HB0801 transformed with plasmid pCT-CT9.55, which encode wild type MbovP475 | Constructed in this study |
| CT9.55_W^246^ | T9.55 transformed with plasmid pCT-CT9.55_W^246^-A, which encode mutated MbovP475 | Constructed in this study |
| CT9.55_^242^NI^243^ | T9.55 transformed with plasmid pCT-CT9.55_^242^NI^243^-AA, which encode mutated MbovP475 | Constructed in this study |
| CT9.55_^242^NI^243^-NA | T9.55 transformed with plasmid pCT-CT9.55_^242^NI^243^-NA, which encode mutated MbovP475 | Constructed in this study |
| CT9.55_^242^NI^243^-NS | T9.55 transformed with plasmid pCT-CT9.55_^242^NI^243^-NS, which encode mutated MbovP475 | Constructed in this study |
| CT9.55_^242^NI^243^-NG | T9.55 transformed with plasmid pCT-CT9.55_^242^NI^243^-NG, which encode mutated MbovP475 | Constructed in this study |
| *E. coli* DH5α | Purchase form TransGen Biotech |  |
| *E. coli* BL21 | Purchase form TransGen Biotech |  |

**Table S3.** Oligonucleotide primers used in the present study

| **Primers** | **Sequences (5’ - 3’)** | **Purpose** |
| --- | --- | --- |
| 475-F | ATGGCTGATATCGGATCCATGAAGCGGAAATTTAGCCT | Amplification of truncated MovP475 without predicted DNA-binding region |
| 475-R | CGAGTGCGGCCGCAAGCTTTTATTTATTACCGATGCTCACTTTC |  |
| 475-∆DB1-R1 | TACAGATATTTCAGATTACCGTCGAACAGTTGGG |  |
| 475-∆DB1-F2 | TGTTCGACGGTAATCTGAAATATCTGTATCGCAC |  |
| 475-∆DB2-R1 | ACCCGGTCTTGTCTCCAACATCCCAGTTGCTTAT |  |
| 475-∆DB2-F2 | GCAACTGGGATGTTGGAGACAAGACCGGGTATGA |  |
| 475-∆DB3-R | CGAGTGCGGCCGCAAGCTTTTAATACCCGGTCTTGTCTC |  |
| 475-W246-R1 | GCTCACGTCTGCTTTGCTAAT |  |
| 475-W246-F2 | GTAATATTAGCAAAGCAGACGTGA |  |
| 475-M254-R1 | ATATCATGTGCATTGGTCACG |  |
| 475-M254-F2 | CGTGACCAATGCACATGATAT |  |
| 475-F258-R1 | CACCATTTGCCATATCATGCAT |  |
| 475-F258-F2 | ATGCATGATATGGCAAATGGTG |  |
| ILDR1-F | GAGGAGGAGAAGGAGAGGCA | Detection of the transcription level of genes regulated by MbovP475 |
| ILDR1-R | CGCCTCTTCACACTCCCTTT |  |
| AMIGO1-F | AGATTGAGCATGGGGGTGTG |  |
| AMIGO1-R | TGGCTGGAAGGTTTCTCGAC |  |
| MCF2L2-F | AAACGTTGCTCAAGCAGGGA |  |
| MCF2L2-R | GCCCTTTTTCTCAAGCAGGAA |  |
| RBM17-F | TTTTCCAGGCGACCTGATCC |  |
| RBM17-R | CATGTTGGCGAGGAAGGAGT |  |
| HMGB1-F | TCCCAATGCACCTAAGAGGC |  |
| HMGB1-R | TCCGCAGCAGTGTTATTCCA |  |
| IGFBP7-F | GCAAGGTCCTTCCATCGTGA |  |
| IGFBP7-R | GCCTGTCCTTGGGAATTGGA |  |
| CRYAB-F | TCCTCTGATGGGGTCCTCAC |  |
| CRYAB-R | ATCTACTTCTTGGGGGCTGC |  |
| β-actin-F | AGCAAGCAGGAGTACGATGAG |  |
| β-actin-R | ATCCAACCGACTGCTGTCA |  |
| 5’C6-Biotin-CRYAB-F | TCTCTTCCCAGCTTCTACTTCCCTGAGCCCCTTCTACCTTCGGCCGCCCTCATTTCTGCG | Preparation of promoter fragment (+108 bp to +167 bp from TSS) |
| 5’C6-Biotin-CRYAB-R | CGCAGAAATGAGGGCGGCCGAAGGTAGAAGGGGCTCAGGGAAGTAGAAGCTGGGAAGAGA |  |
| 5’C6-Biotin-MCF2L2-F | CAACTAACACTTTACGGGAGTGGGCACATCATTCCTGAATAAGGACACGCGCGTGCGCAC | Preparation of promoter fragment (+53 bp to +112 bp from TSS) |
| 5’C6-Biotin-MCF2L2-R | GTGCGCACGCGCGTGTCCTTATTCAGGAATGATGTGCCCACTCCCGTAAAGTGTTAGTTG |  |
| 5’C6-Biotin- CRYAB-CF | ATAAAACTCCTGACATCACCATTCCAGAAGCTTCACAAGACTGCGTATATAAGGGGCTGG | Preparation of promoter fragment (-120 bp to -61 bp from TSS) |
| 5’C6-Biotin- CRYAB-CR | CCAGCCCCTTATATACGCAGTCTTGTGAAGCTTCTGGAATGGTGATGTCAGGAGTTTTAT |  |
| CRYAB siRNA | GCACCUGUUGGAGUCUGAUTT | CRYAB and MCF2L2 knockdown |
| MCF2L2 siRNA | CCUUAUACAGAGACAGAUUTT |  |
| NC siRNA | UUCUUCGAACGUGUCACGUTT |  |
| NI-F | TTTTGATGGCGCTGCTTCAAAATGAGATGTTTC | Construct pCT-CT9.55_^242^NI^243^ |
| NI-R | AGTTGTGTATAGCTAAATAAG |  |
| W-F | CATTTCAAAAGCTGATGTTTCAAGTGTTACAAATATG | Construct pCT-CT9.55_W^246^ |
| W-R | TTGCCATCAAAAAGTTGTG |  |
| NA-F | TGATGGCAACGCTTCAAAATGAGATG | Construct pCT-CT9.55_^242^NI^243^-NA, pCT-CT9.55_^242^NI^243^-NS, and pCT-CT9.55_^242^NI^243^-NG |
| NS-F | TGATGGCAACAGTTCAAAATGAG |  |
| NG-F | TGATGGCAACGGTTCAAAATGAGATGTTTC |  |
| N-R | AAAAGTTGTGTATAGCTAAATAAG |  |
| MCF2L2-PF | TCTCTATCGATAGGTACCCTGCACAGCCCGCGCTCCC | Construct promoter-pGL3-Basic (MCF2L2) |
| MCF2L2-PR | TAGATCGCAGATCTCGAGCTACGCCGTCTCACAGCGCGTC |  |
| AMIGO1-PF | TCTCTATCGATAGGTACCGGAACGTGACTAATGAATTTCCCG | Construct promoter-pGL3-Basic (AMIGO1) |
| AMIGO1-PR | TAGATCGCAGATCTCGAGCTGCTCGGCGAGGACGTTTC |  |
| CRYAB-PF | TCTCTATCGATAGGTACCATGCTGGTGGTCACTGCTC | Construct promoter-pGL3-Basic (CRYAB) |
| CRYAB-PR | TAGATCGCAGATCTCGAGCTCTGAGAGGCCAGTGTCAATC |  |
| RBM17-PF | TCTCTATCGATAGGTACCTTCACTTCACACAGTTAATTCAGCACT | Construct promoter-pGL3-Basic (RBM17) |
| RBM17-PR | TAGATCGCAGATCTCGAGCTAAAGCCGCGGCCTCACC |  |
| HMGB1-PF | TCTCTATCGATAGGTACCCGTGTCGCTCTCACTTTTGAAGGT | Construct promoter-pGL3-Basic (HMGB1) |
| HMGB1-PR | TAGATCGCAGATCTCGAGCTCATTTGCCTTTGTGTGGATCCT |  |
