# Supplementary figures and images for: Novel mycoplasma nucleomodulin MbovP475 decreased cell viability by regulating expression of CRYAB and MCF2L2
Source: Virulence. 2022 Sep 19;13(1):1590–613. doi: 10.1080/21505594.2022.2117762 (PMC9487752; doi:10.1080/21505594.2022.2117762)

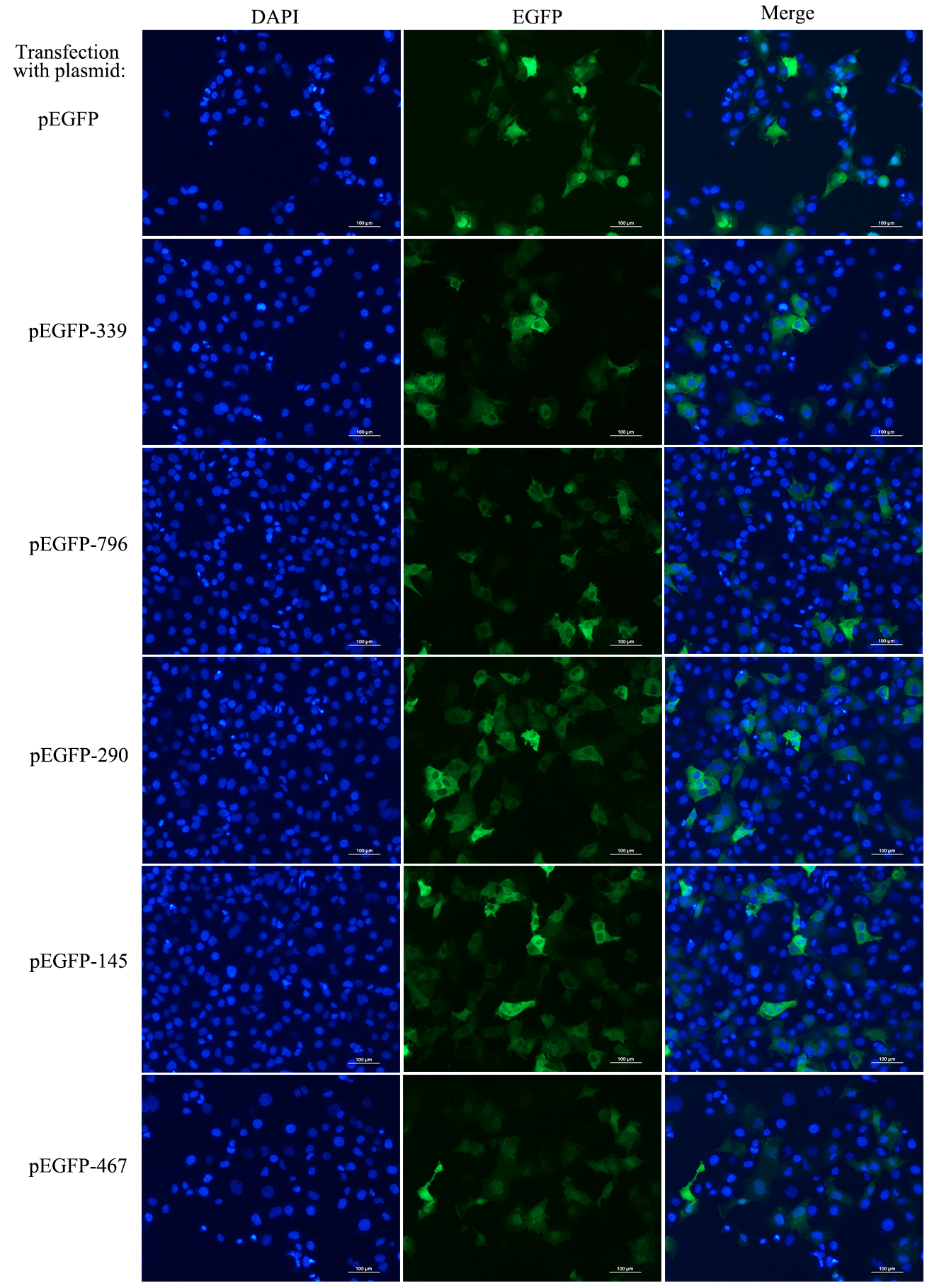

Supplement: Supplemental Material [file KVIR_A_2117762_SM4774.zip › supplementary/Fig.S1.tif]

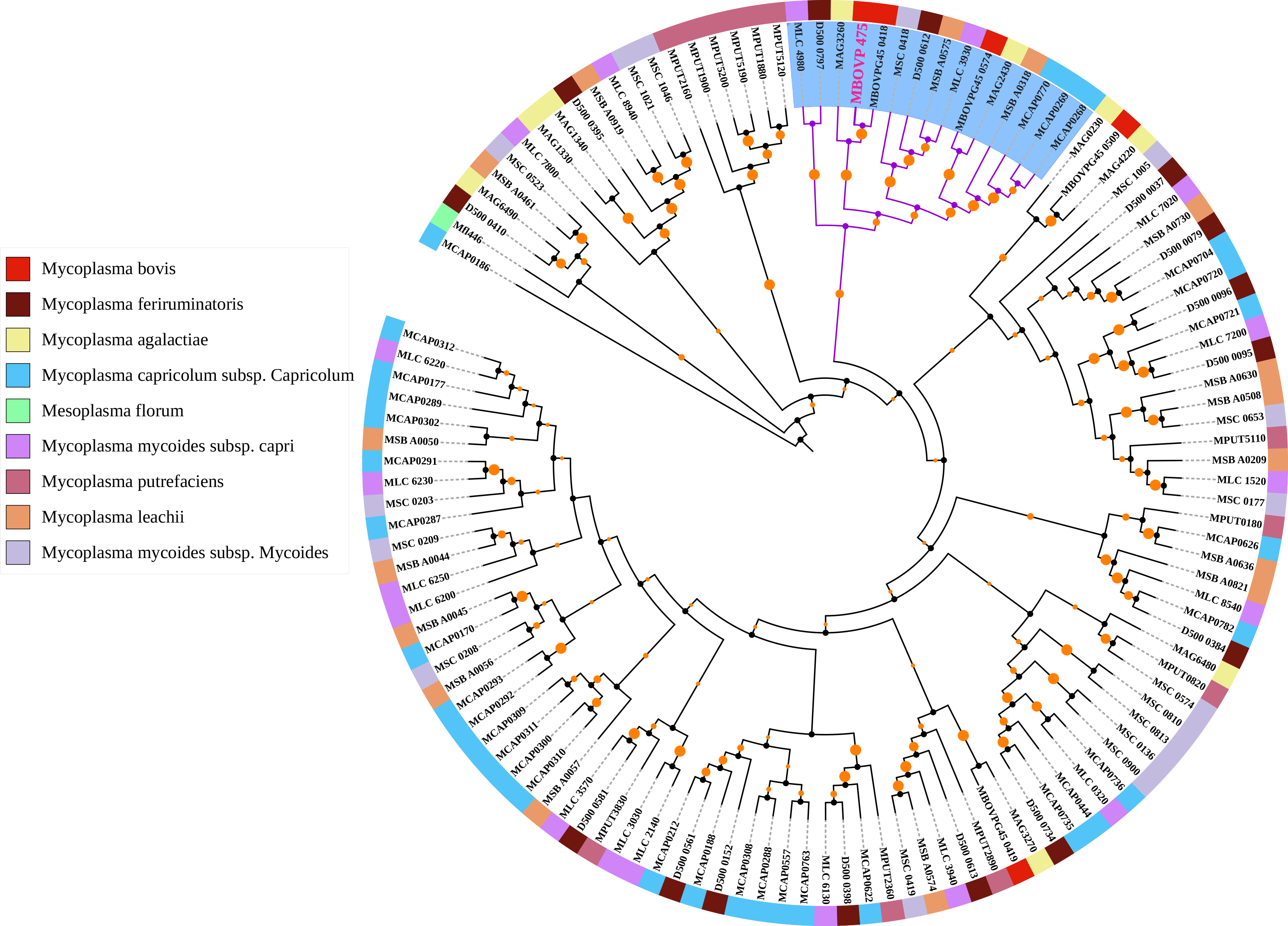

Supplement: Supplemental Material [file KVIR_A_2117762_SM4774.zip › supplementary/Fig.S2.tif]

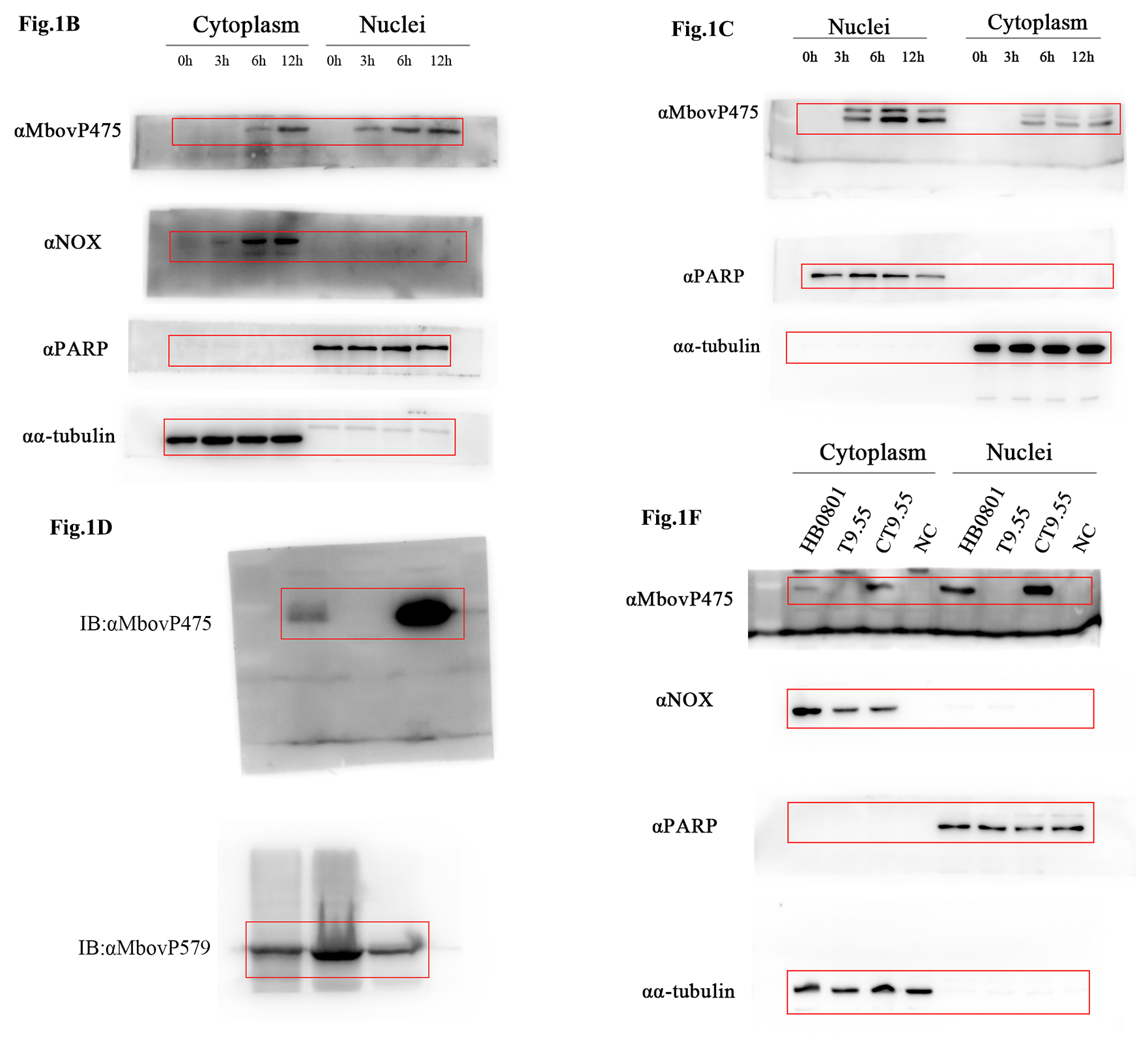

Supplement: Supplemental Material [file KVIR_A_2117762_SM4774.zip › supplementary/Fig.S3.tif]

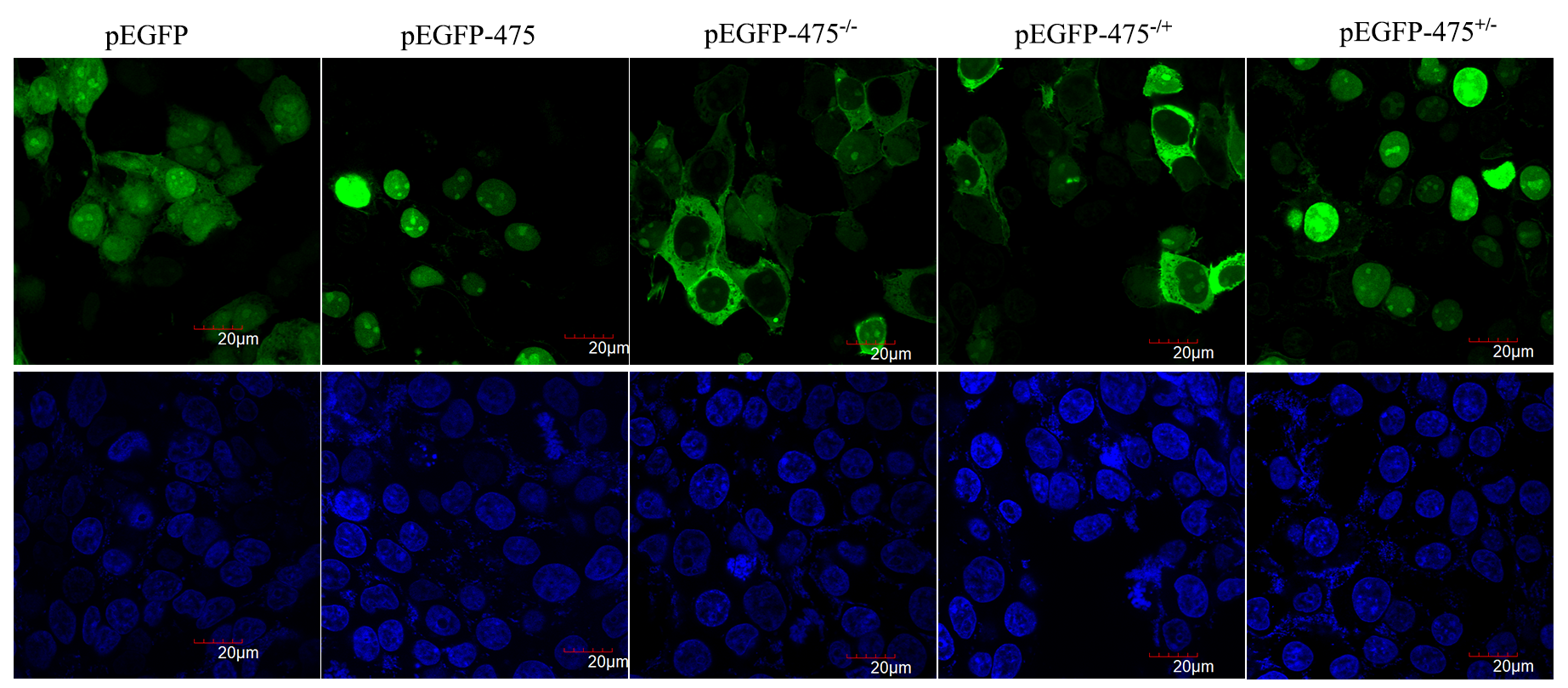

Supplement: Supplemental Material [file KVIR_A_2117762_SM4774.zip › supplementary/Fig.S4.tif]

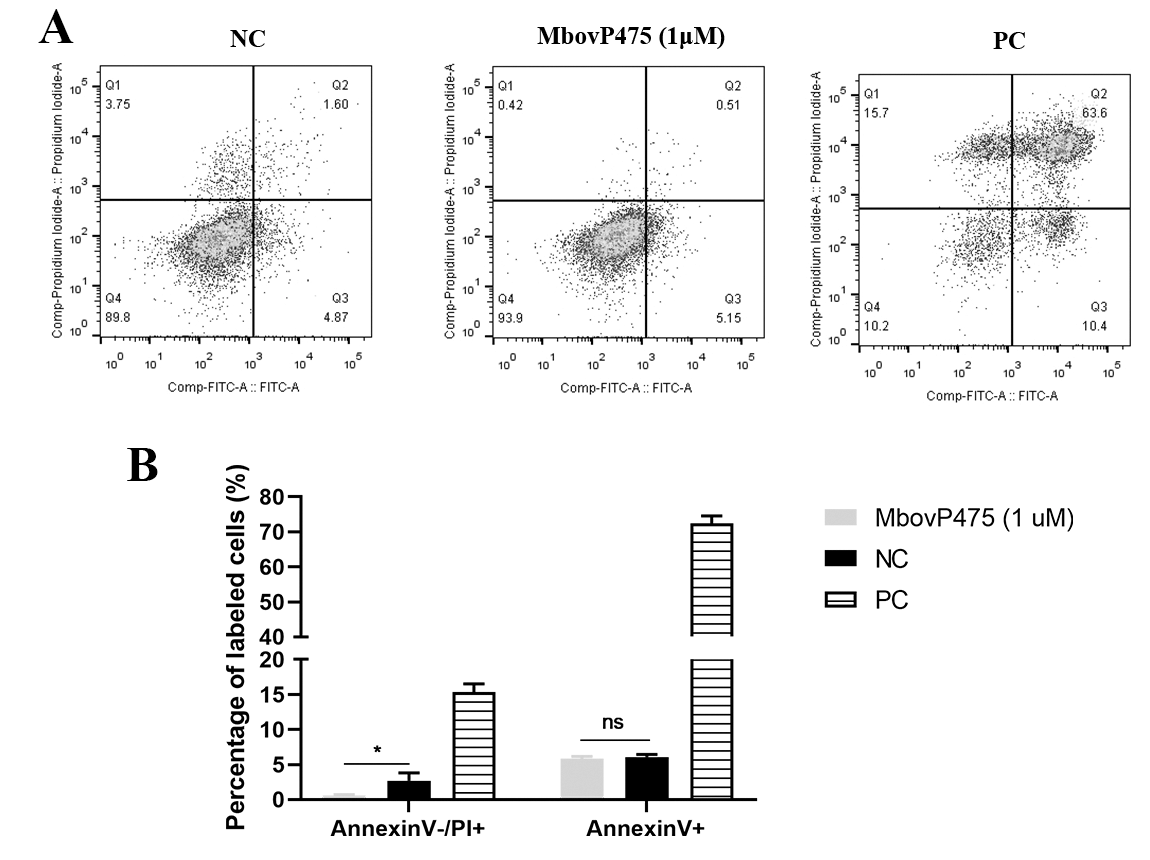

Supplement: Supplemental Material [file KVIR_A_2117762_SM4774.zip › supplementary/Fig.S5.tif]

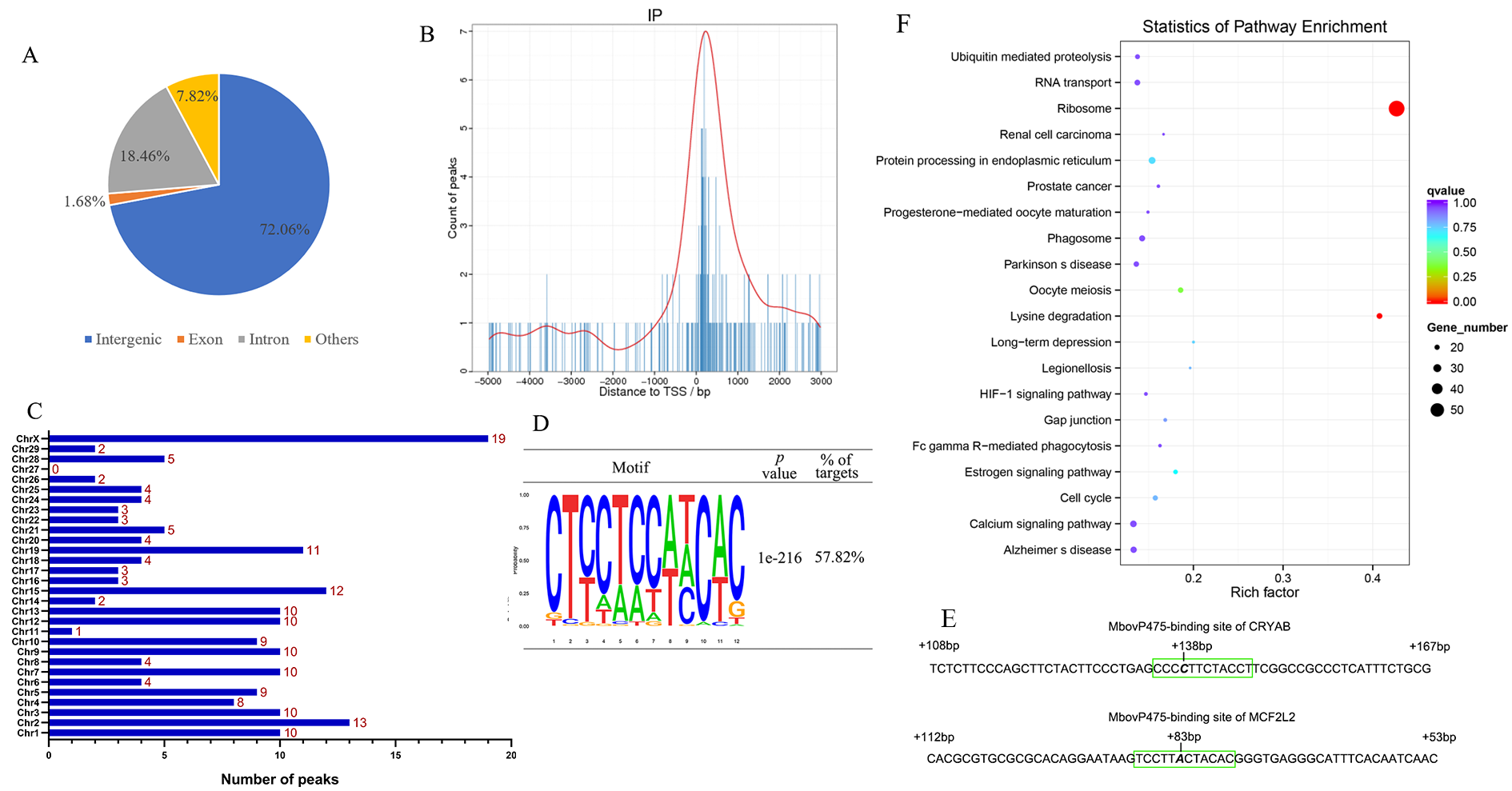

Supplement: Supplemental Material [file KVIR_A_2117762_SM4774.zip › supplementary/Fig.S6.tif]

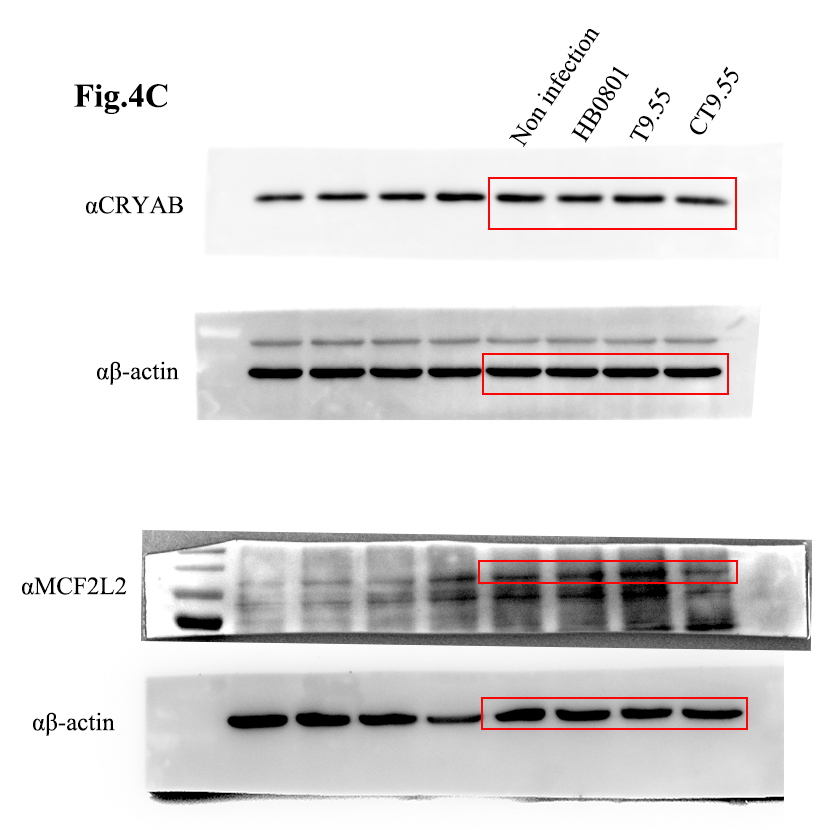

Supplement: Supplemental Material [file KVIR_A_2117762_SM4774.zip › supplementary/Fig.S7.tif]

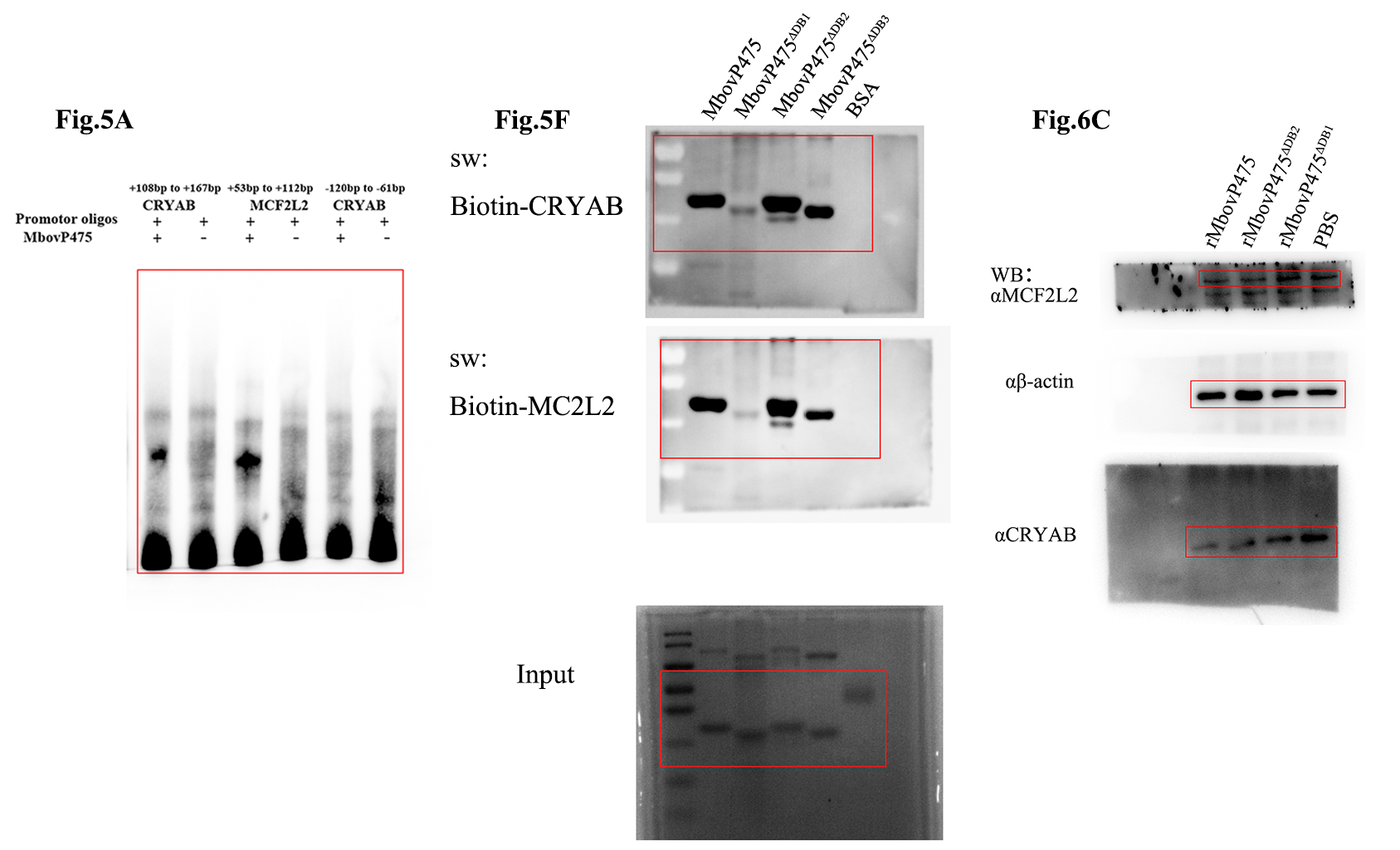

Supplement: Supplemental Material [file KVIR_A_2117762_SM4774.zip › supplementary/Fig.S8.tif]

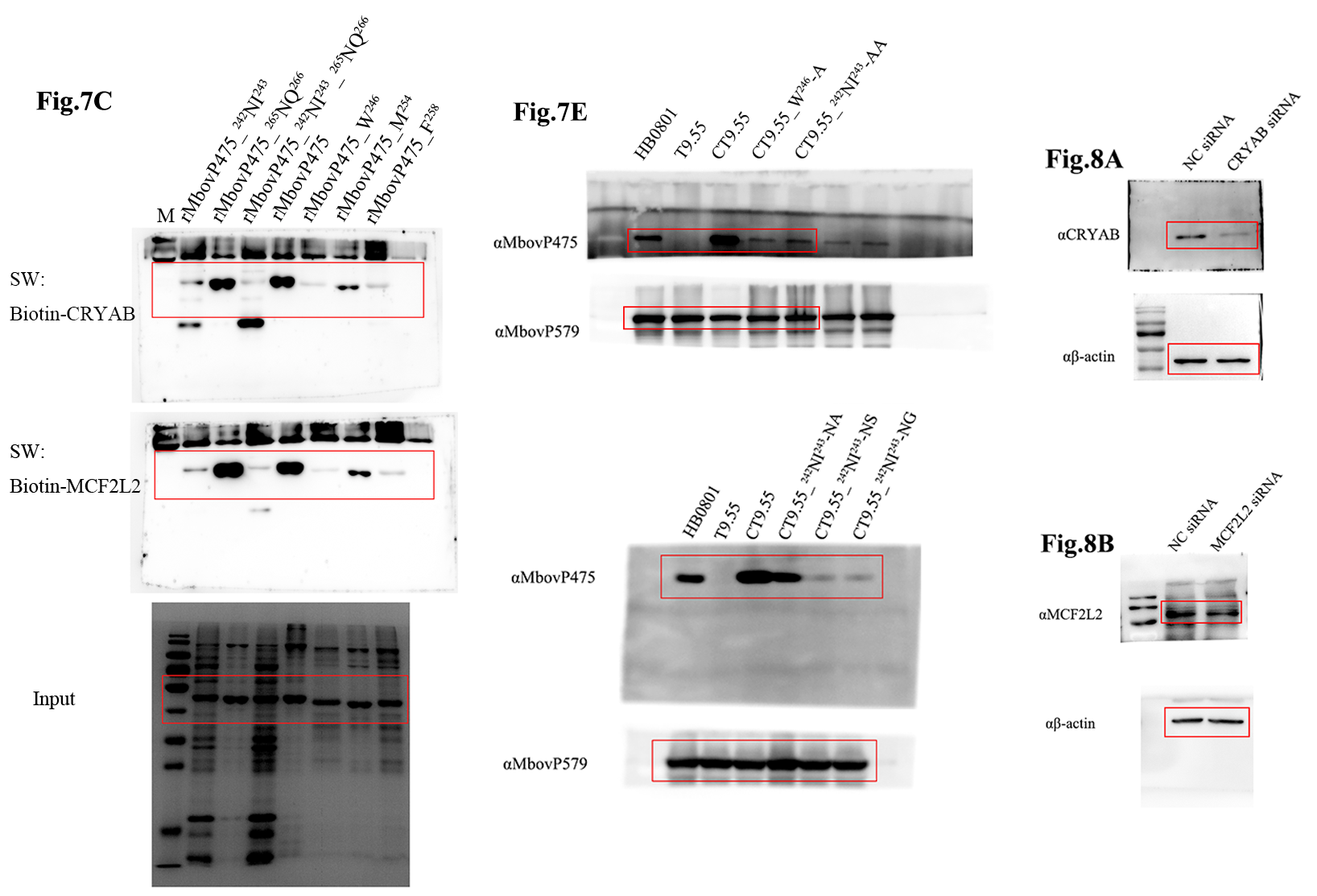

Supplement: Supplemental Material [file KVIR_A_2117762_SM4774.zip › supplementary/Fig.S9.tif]
